# A tutorial review on solid oxide fuel cells: fundamentals, materials, and applications

**DOI:** 10.1007/s11581-024-05824-7

**Published:** 2024-10-07

**Authors:** Daniel Sikstrom, Venkataraman Thangadurai

**Affiliations:** 1 Department of Chemistry, University of Calgary, 2500 University Dr NW, Calgary, Alberta T2N 1N4 Canada; 2 School of Chemistry, University of St Andrews, St Andrews, Fife, KY16 9ST UK

**Keywords:** Solid oxide fuel cells, Cathode, Anode, Electrolyte, Interconnector, Efficiency

## Abstract

Solid oxide fuel cells (SOFCs) are recognized as a clean energy source that, unlike internal combustion engines, produces no CO_2_ during operation when H_2_ is used as a fuel. They use a highly efficient chemical-to-electrical energy conversion process to convert oxygen and hydrogen into electricity and water. They can provide smaller-scale power for transportation (e.g., cars, buses, and ships) and be scaled up to provide long-term energy for an electrical grid, making SOFCs a promising, clean alternative to hydrocarbon combustion. Conventional SOFCs faced challenges of high operating temperatures, high cost, and poor durability. Research into advanced cathode, anode, electrolyte, and interconnect materials is providing more insight into the ideal structural and chemical properties that enable the commercialization of highly stable and efficient intermediate temperature (IT) SOFCs. In this paper, we discuss the functional properties of the cathode, anode, electrolyte, and interconnectors for IT-SOFCs. The performance of SOFCs depends not only on the materials used but also on the optimization of operating conditions to maximize efficiency. The voltaic, thermodynamic, and fuel efficiency of SOFCs is presented.

## Introduction

**Fig. 1:**
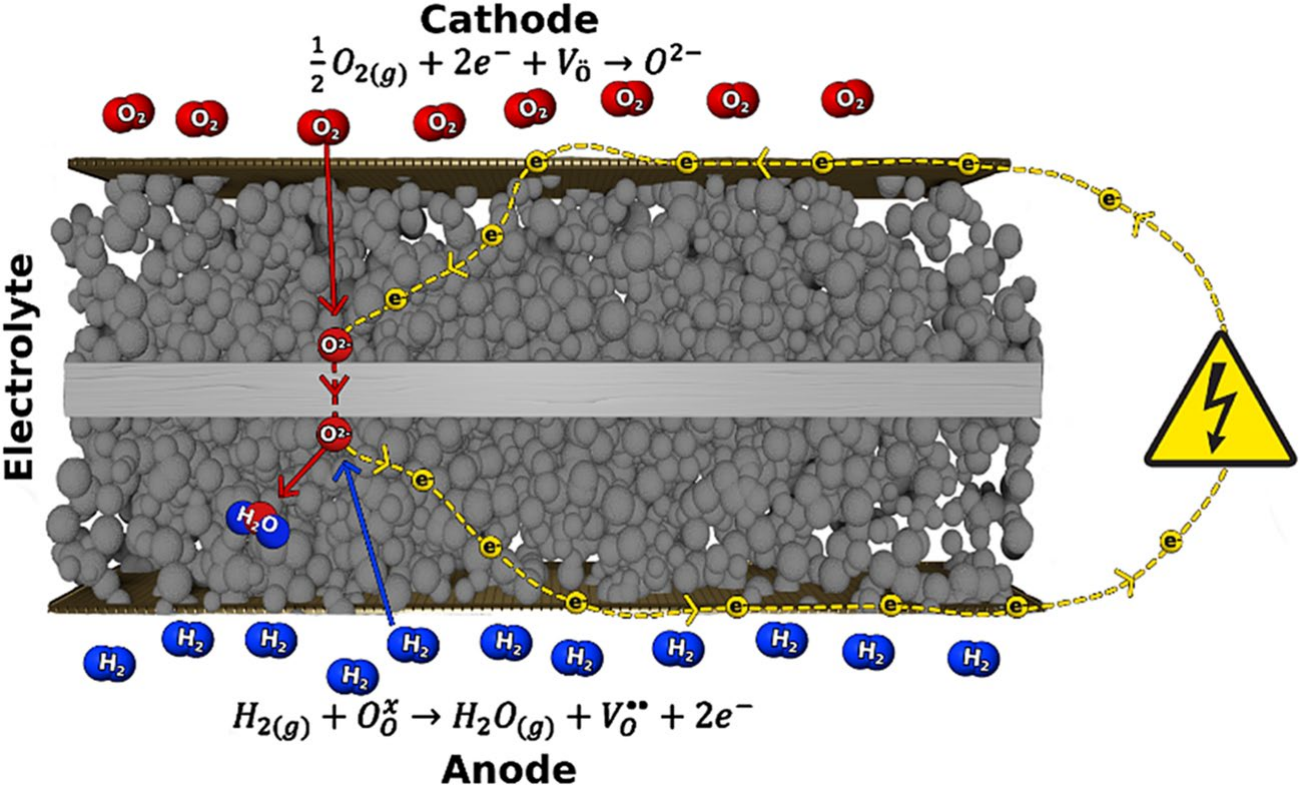
Schematic diagram of a solid oxide fuel cell

To meet global energy demands, while achieving net-zero emissions by 2050, it is essential to advance environmentally sustainable alternatives to the combustion of fossil fuels [1]. Solid oxide fuel cells (SOFCs) are a promising alternative that uses a highly efficient chemical-to-electrical energy conversion process to convert oxygen and hydrogen into electricity and water (Fig. 1) [2]. Compared to other fuel cells, such as proton exchange membrane, alkaline, or direct methanol fuel cells, SOFCs can achieve the highest efficiency and power output [3]. Despite the efficiency of SOFCs, they have yet to see widespread distribution due to the high cost of manufacturing and poor durability. Recent focus has been on developing alternative cathode, electrolyte, and anode materials that can operate at intermediate temperatures (IT) (600–800 °C) to allow for the use of cheaper interconnect materials and improve the durability [4]. Here, we discuss the recent development of cathodes, electrolytes, anodes, and interconnects for IT SOFCs, as well as the chemical and physical requirements. Furthermore, the thermodynamic, fuel, and voltaic efficiency of SOFCs for optimal cell performance is suggested.

## Solid oxide fuel cell components

### Cathodes

**Fig. 2:**
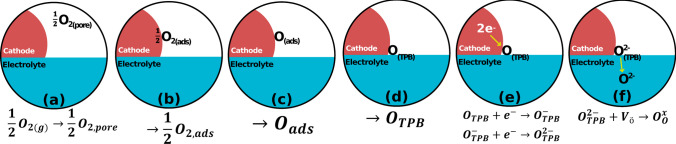
Proposed mechanism for the ORR in electronic conducting cathodes for SOFCs. **a** Diffusion of O_2_ into the pores of the cathode, **b** adsorption of O_2_ onto the cathode surface, **c** dissociation of adsorbed O_2_, **d** diffusion of O to the TPB, **e** electron transfer, and **f** oxygen-ion transfer into a vacancy in the ion conductor

**Fig. 3:**
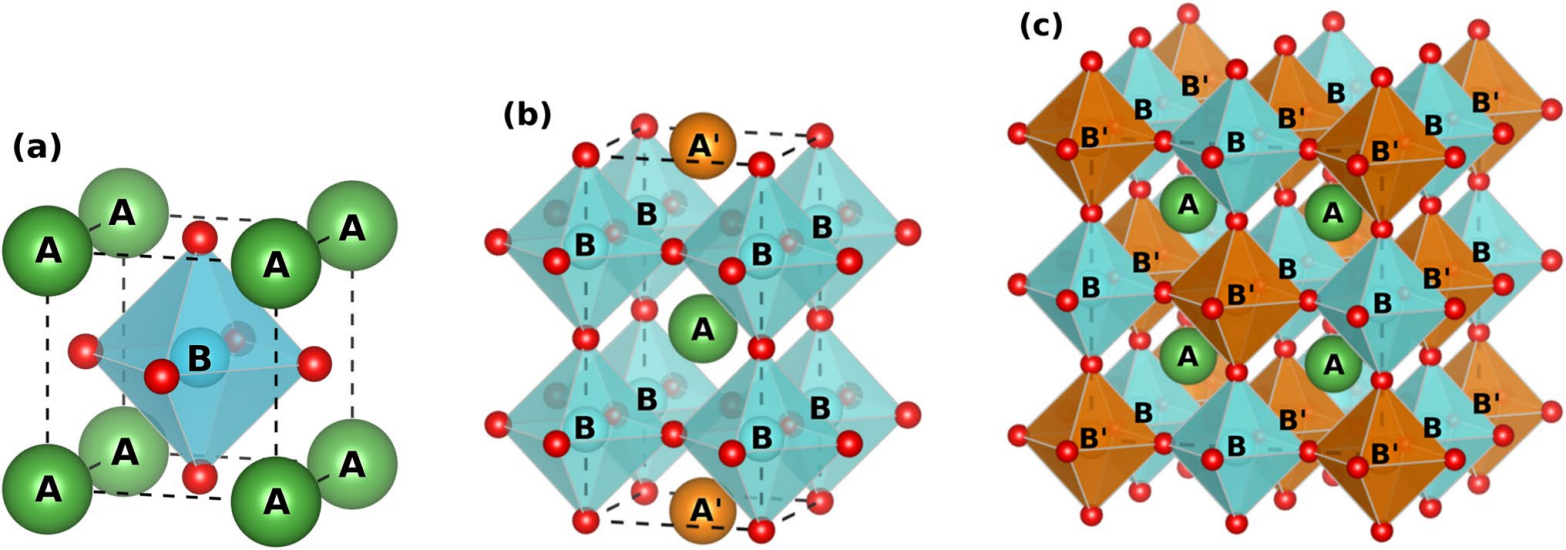
**a** The generalized crystal structure of an ABO_3−*δ*_ perovskite in the *Pm3-m* space group (No. 221); the A-site at the 4*a*-Wyckoff positions; the B-site at the 4*b*-Wyckoff positions; and the oxygen site at the 8c-Wyckoff position. **b** The generalized crystal structure of an AA’B_2_O_5−*δ*_ layered perovskite in the *P4* space group (No. 75); the A and A’-site at the 1b-Wyckoff positions; the B-site at the 1a-Wyckoff positions; and the oxygen site at the 1a and 2c-Wyckoff positions, respectively. **c** The generalized crystal structure of an A_2_BB’O_6−*δ*_ double perovskite in the Fm3-m space group (No. 225); the A-site at the 8c-Wyckoff position; the B-site at the 4b-Wyckoff position; the B’-site at the 4a-Wyckoff position; and the oxygen at the 24e-Wyckoff position

Cathode materials for SOFCs are designed to meet the following functional properties. (i) High catalytic activity towards the oxygen reduction reaction (ORR) at intermediate temperatures (IT) (600–800 °C). The ORR is a multistep reaction involving oxygen diffusion, adsorption, dissociation, diffusion towards the catalytically active triple-phase-boundary (TPB), electron transfer, and ion transfer to the electrolyte (Fig. 2) [5]. For a material to have high catalytic activity towards the ORR, it must have low resistance for each step of the ORR; the total resistance for the ORR should be less than 0.1 Ω cm^2^ at an IT for high-performance SOFCs. This is experimentally determined using electrochemical impedance spectroscopy (EIS); the total resistance can be deconvoluted into the individual steps of the ORR using a distribution function of relaxation times (DRT) and equivalent circuit modeling [6–8].

(ii) High total electrical conductivity (mixed ionic and electronic conductivity (MIEC)) (> 100 S/cm at ITs in air/O_2_) to improve the electron charge transfer kinetics of the ORR and to extend the TPB. The conductivity of an SOFC cathode is measured using a 2 or 4-probe DC or AC technique [9]. By using a ionic conducting probe such as Sm-doped CeO_2_ and Y-doped ZrO_2_ (electron-blocking electrode), the ionic conductivity of the cathode can be isolated [10]. In contrast, by using a purely electronic conducting probe such as dense Pt (ion-blocked), the electronic conductivity of the material can be isolated [11]. Interface compatibility should be considered carefully, and measurements should be performed using different sample dimensions to ensure reproducibility. (iii) Compatibility with the electrolyte and interconnect materials including chemical stability and a matching thermal expansion coefficient (TEC). The chemical stability can be determined using X-ray diffraction (XRD), scanning electron microscopy (SEM), and elemental mapping (EDS) of the samples before and after high-temperature exposure to the electrolyte [7, 12]. The TEC is determined by measuring the samples displacement (either length or volume) as a function of temperature; the acceptable limit for a TEC mismatch between the cathode and electrolyte/interconnects is ~ 15–20% to avoid cell cracking and cathode delamination [13]. (iv) Structural stability under SOFC operating conditions (IT, oxidizing environment, dual-atmosphere, and polarization). The chemical stability of SOFC components can be determined using microscopy and spectroscopy methods before and after operating [14]. EIS of the SOFC during operation and analyzing how the spectrum changes with time using DRT gives insight into how the structural changes are affecting the ORR mechanism [15–17]. For example, an increase in the high-frequency impedance (charge transfer resistance) may indicate reactivity between the cathode and electrolyte [18]. SOFC cathodes that can meet these criteria include perovskites, layered perovskites, double perovskites, and Ruddlesden-Popper phase materials (Fig. 3 and Table 1) [6–8, 19–34].

**Table 1:** Typical cathodes for SOFCs and their properties [6–8, 19–34]

Material	Area-specific resistance (Ω cm^2^) (air)	Total conductivity (S/cm)	Thermal expansion coefficient (10^−6^/K)	Structure type
La_0.8_Sr_0.2_MnO_3−*δ*_	0.3 (700 °C) [19]	150 (700 °C, air) [19]	11.8 [20]	Perovskite
La_0.6_Sr_0.4_CoO_3−*δ*_	0.02 (600 °C) [21]	2000 (650 °C, pO_2_ = 0.1) [22]	19.6 [23]	Perovskite
La_0.6_Sr_0.4_Co_0.2_Fe_0.8_O_3−*δ*_	0.21 (600 °C) [24]	431 (650 °C, air) [25]	13.3 [26]	Perovskite
Ba_0.5_Sr_0.5_Co_0.8_Fe_0.2_O_3−*δ*_	0.035 (700 °C) [27]	15 (700 °C, air) [28]	19.2 [29]	Perovskite
GdBaCo_2_O_5+*δ*_	0.13 (700 °C) [30]	450 (600 °C, air) [31]	~ 10 [32]	Layered-perovskite
Ba_2_CoMo_0.5_Nb_0.5_O_6−*δ*_	0.2 (700 °C) [33]	1 (700 °C, air) [33]	16 [33]	Double-perovskite
Nd_0.75_Ba_0.25_Co_0.8_Fe_0.2_O_3−*δ*_	0.11 (750 °C) [6]	674 (700 °C, air) [6]	-	Perovskite
Nd_0.3_Sr_0.7_Co_0.8_Fe_0.2_O_3−*δ*_	0.1 (750 °C) [7]	300 (700 °C, air) [7]	-	Perovskite
PrSrCoFeO_5+*δ*_	0.07 (750 °C) [8]	500 (700 °C, air) [8]	-	Layered-perovskite
SrCo_1.6_Fe_0.4_O_6−*δ*_	0.25 (700 °C) [34]	6 (700 °C, air)[34]	-	Double-perovskite

#### Perovskite-type cathodes

**Fig. 4:**
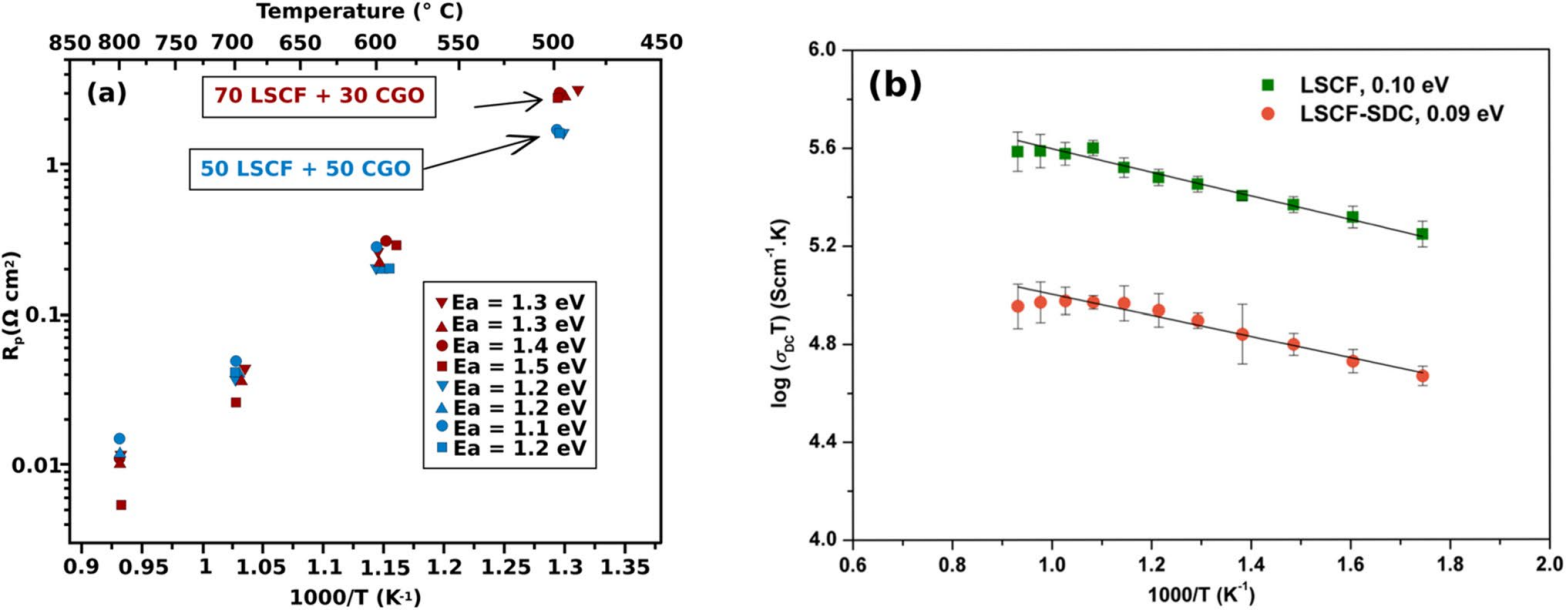
**a** Arrhenius plot of the polarization resistance measured at different temperatures for a LSCF/CGO composite cathode (70 wt%/30 wt% and 50 wt%/50 wt%) on a CGO electrolyte (reproduced from Wang and Mogensen (2005)) [43]. **b** Arrhenius plot of the conductivity measured at different temperatures for a LSCF and a LSCF/SDC composite cathode (50 wt%/50 wt%) [25]

**Fig. 5:**
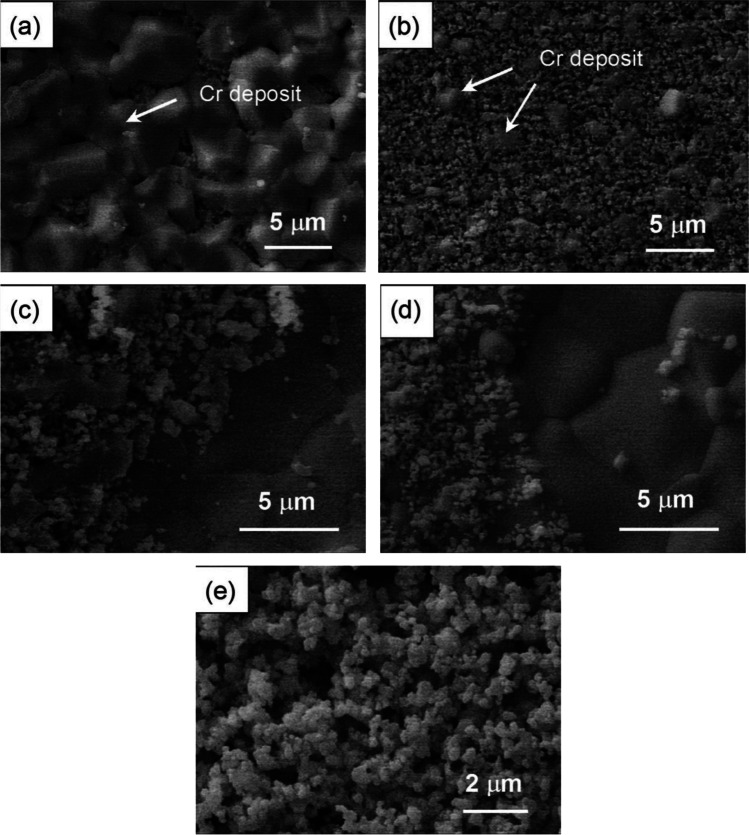
SEM micrographs of a LSCF electrode after polarization at 200 mA cm^−2^ and 900 °C in the presence of Fe–Cr alloy for 20 h. **a** Electrode surface under the rib of interconnect; **b** electrode surface under the channel of interconnect; **c** the edge of the LSCF electrode under the rib of interconnect; and **d** the edge of the LSCF electrode under the channel of interconnect. The surface of a LSCF electrode after polarization at 200 mA cm.^−2^ and 900 °C for 2 h in the absence of Fe–Cr alloy is shown in **e** [46]

Perovskites are materials that share the same crystal structure as the mineral CaTiO_3_ giving them the general chemical formula of ABO_3_; the larger size atom sits at the 12-coordinate A-site while the smaller size atom sits at the 6-coordinate B-site (Fig. 3) [35]. The structure is formed by connecting BO_6_ octahedrons with the A-ion occupying the space between the octahedrons [36]. Doping lower oxidation state ions into the A and B site introduces oxygen vacancies which improve the kinetics of the last step of the ORR (Fig. 2) [37, 38]. If the cathode is a mixed ionic and electronic conductors (MIECs), this step can occur at the cathode-oxygen interface; for purely electronic conducting cathodes, this step can only occur at the TPB [39]. Lanthanum strontium manganite (LSM) has been extensively studied owing to its high stability and catalytic activity towards the ORR at elevated temperatures (< 0.1 Ω cm^2^ at 800 °C) [2, 40]. However, at ITs, the resistance at the LSM becomes far to large (> 10 Ω cm^2^ at 650 °C) to be useful in SOFCs operating at ITs [41]. LSM also has negligible ionic conductivity (< 6 × 10^−7^ S/cm) reducing the active area for ORR [42]. Lanthanum strontium cobalt ferrite (LSCF) is one of the most popular cathodes for IT SOFCs due to its MIEC properties, high electrical conductivity (> 300 S/cm at 700 °C), and ORR catalytic activity (< 0.1 Ω cm^2^ at 700 °C) (Fig. 4) [25, 43]. However, LSCF has been shown to become poisoned by Cr-containing interconnects and experiences Sr segregation during SOFC operating conditions (Fig. 5) [44–46].

#### Layered perovskite-type cathodes

**Fig. 6:**
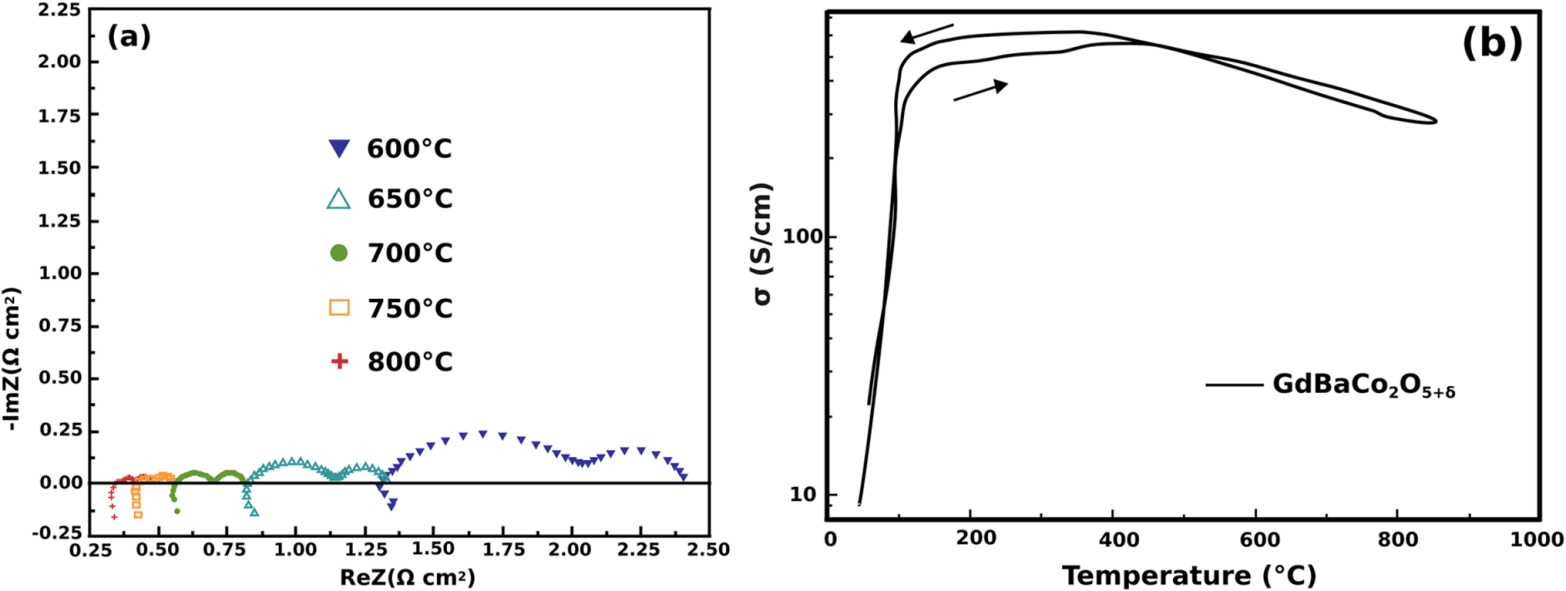
**a** Impedance spectra for a GBCO cathode at various temperatures. **b** Conductivity as a function of the temperature for GBCO (reproduced from Li et al. (2008)) [31]

**Fig. 7:**
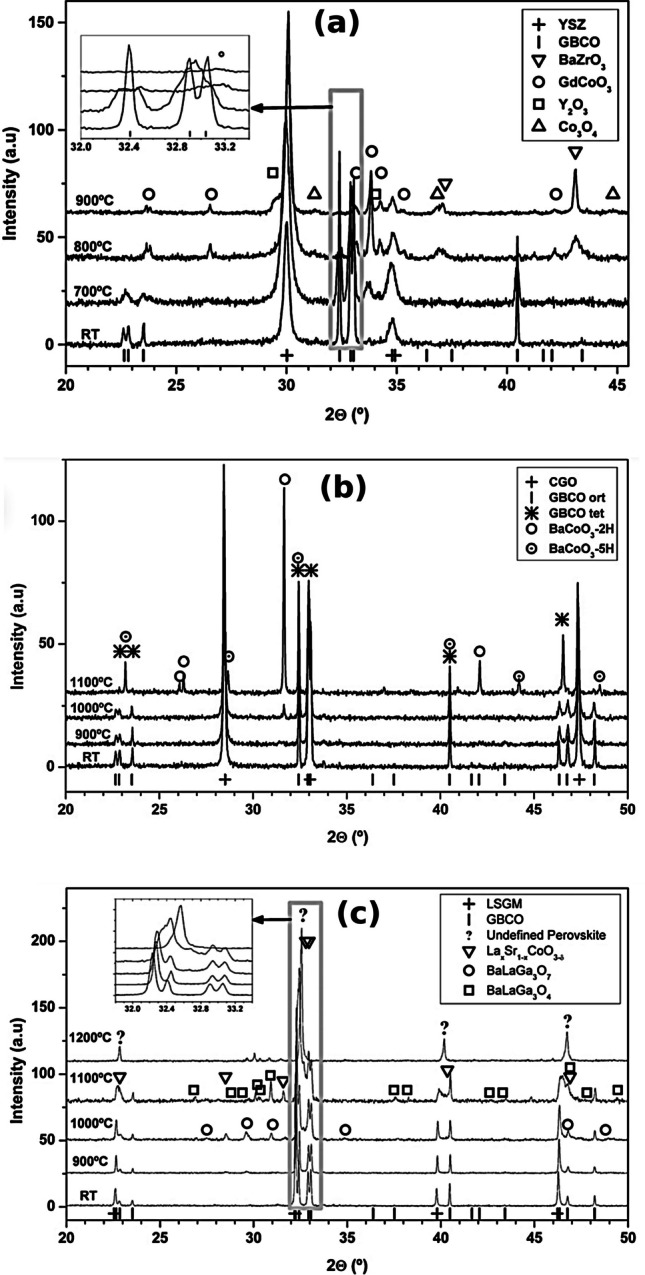
**a** XRD patterns corresponding to mixtures of 50 wt% GdBaCo_2_O_5+*δ*_ (GBCO) and 50 wt% YSZ powders at room temperature and after calcined at 700, 800, and 900 °C for 24 h in air. **b** XRD patterns corresponding to mixtures of 50 wt% GBCO and 50 wt% CGO powders at room temperature and after calcined at 900, 1000, and 1100 °C for 24 h in air. **c** XRD patterns corresponding to mixtures of 50 wt% GBCO and 50 wt% LSGM powders at room temperature and after calcined at 900, 1000, 1100, and 1200 °C for 24 h in air [51]

Layered perovskites are described by the general chemical formula AA’B_2_O_5+*δ*_ with A being lanthanides or yttrium; A’ being Ba or Sr; and B being Co, Fe, or Mn (Fig. 3) [47]. Their structure has ordered A-site cations where the A and A’ layers alternate along the (001) plane and oxygen vacancies appear in the A layer [47, 48]. Interest in these materials as potential cathodes for SOFCs is owed to their low TECs and high MIEC with a more stable B site when compared to disordered perovskites [49]. Among the layered perovskites, GdBaCo_2_O_5+*δ*_ (GBCO) shows a total conductivity of 100–1000 S/cm, ionic conductivity of 0.01 S/cm, and ORR catalytic activity of 0.1 Ω cm^2^ at 700 °C (Fig. 6) [30, 31, 50]. However, GBCO has shown to be highly reactive with yttrium-stabilized zirconia (YSZ) electrolyte as well as forming secondary phases with La_0.8_Sr_0.2_Ga_0.8_Mg_0.2_O_3−*δ*_ (LSGM) and Ce_0.9_Gd_0.1_O_1.95_ (CGO) electrolytes which may reduce the cells performance with time (Fig. 7) [51]. Further research still needs to be done to determine the structural stability of layered perovskite-type cathodes under working SOFC conditions [51, 52].

#### Double-perovskite-type cathodes

**Fig. 8:**
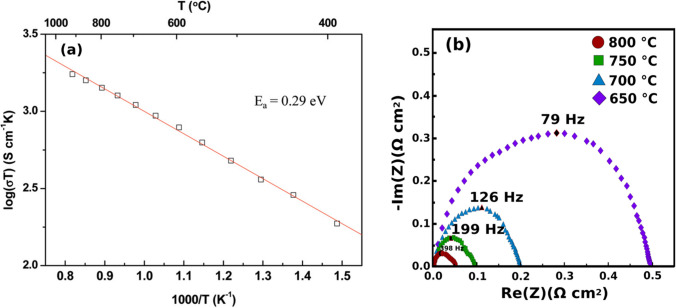
**a** Temperature dependence of the DC electrical conductivity for Ba_2_CoMo_0.5_Nb_0.5_O_6−*δ*_ (BCMN) samples in air. **b** Cathode polarization of BCMN on an SDC electrolyte symmetric cell measured in air at 800, 750, 700, and 650 °C. The electrolyte contribution has been subtracted from the overall resistance, which is the cell resistance divided by two and represents the polarization of one electrode (reproduced from Deng et al. (2009)) [33]

Double perovskites are described by the general chemical formula A_2_BB’O_6−*δ*_ or AA’BB’O_6−*δ*_ where alkali, alkaline earth, or rare earth ions sit at the A and A’ site and metal ions sit at the B and B’ site (Fig. 3) [36]. Their structure is similar to that of a single perovskite with BO_6_ and B’O_6_ octahedrons where A and A’ occupy the space between the octahedrons [36]. These materials are interesting candidates for SOFC cathodes due to their ability to hold high oxygen vacancies and stabilize multivalent ions through doping which increases the electronic conductivity [33, 53]. Ba_2_CoMo_0.5_Nb_0.5_O_6−*δ*_ (BCMN) was found to have high catalytic activity towards the ORR (0.1 Ω/cm^2^ at 700 °C), structural stability at ITs (240 h at 750 °C), and no reactivity with the Sm-doped CeO_2_ (SDC) electrolyte [33]. However, the electrical conductivity is very low (1 S/cm at 700 °C) caused by the large separation between multivalent Co-ions (Fig. 8). The separation between the electronically active ions causes low conductivity in other double perovskite cathodes [54–56].

### Anodes

**Fig. 9:**
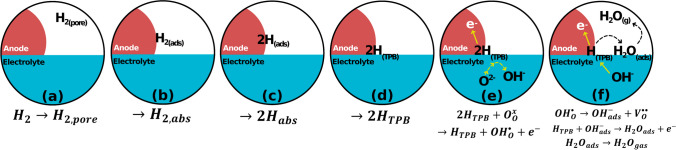
Mechanism for the HOR in a purely electronic conducting anode for SOFCs. **a** Diffusion of H_2_ into the pores of the anode, **b** adsorption of H_2_ onto the anode surface, **c** dissociation of H_2_, **d** diffusion of H to the anode TPB, **e** oxidation of one H at the anode TPB, **f** adsorption of OH^−^ onto the ionic-conductive surface followed by the oxidation of the second H and release of water vapor from the TPB

Anode materials for IT SOFCs are designed to meet the following properties. (i) High catalytic activity towards the hydrogen oxidation reaction (HOR). Similar to ORR, the HOR is also a multistep reaction involving hydrogen diffusion, adsorption, oxidation at the TPB, adsorption of OH^−^ onto the ion conductor’s surface, second oxidation at the TPB, and release of water vapor (Fig. 9) [57]. For a material to have high catalytic activity towards the HOR, it must have low resistance for each step of the HOR; the total resistance for the HOR should be < 0.5 Ω cm^2^ at an IT in H_2_ [58–60]. (ii) High total conductivity (> 100 S/cm) at ITs in an H_2_ environment to improve the electron charge transfer kinetics of the HOR and extend the TPB [61]. (iii) Compatibility with the electrolyte and interconnect materials. (iv) Structural stability under SOFC operating conditions (IT, reducing environment, dual-atmosphere, and polarization). Classes of materials for SOFC anodes that can meet these criteria include Ni-cermet, perovskite-type, and Cu-based anodes (Table 2) [62–72].

**Table 2:** Anodes for SOFCs and their properties [62–72]

Material	Area-specific resistance (Ω cm^2^) (in H_2_)	Total conductivity (S/cm)	Thermal expansion coefficient (10^−6^/K)
Ni-YSZ	0.18 (800 °C) [62]	1809 (800 °C, H_2_) [63]	12.1 [63]
Cu-CeO_2_-ScSZ	0.263 (800 °C) [64]	-	-
La_0.5_Sr_1.5_MnO_4+*δ*_	0.61 (800 °C) [65]	1.5 (800 °C, 3% H_2_) [66]	12.5 [66]
La_0.75_Sr_0.25_Cr_0.5_Mn_0.5_O_3−*δ*_	0.33 (800 °C) [67]	0.95 (800 °C, 5% H_2_) [68]	8.9 [68]
La_0.4_Sr_0.6_Ti_0.4_Mn_0.6_O_3−*δ*_	0.32 (856 °C) [69]	1.4 (800 °C, 4% H_2_) [69]	11.9 [69]
Sr_2_Fe_1.3_Co_0.2_Mo_0.5_O_6−*δ*_	0.29 (800 °C) [70]	2.4 (800 °C, 5% H_2_) [70]	-
Sr_2_FeNi_0.2_Cu_0.2_Co_0.1_Mo_0.5_O_6_	0.32 (800 °C)[71]	-	18.3 [71]
Sr_2_CrMoO_6−*δ*_	0.71 (800 °C) [72]	92 (800 °C, 5% H_2_) [72]	11.2 [72]

#### Ni-cermet anodes

**Fig. 10:**
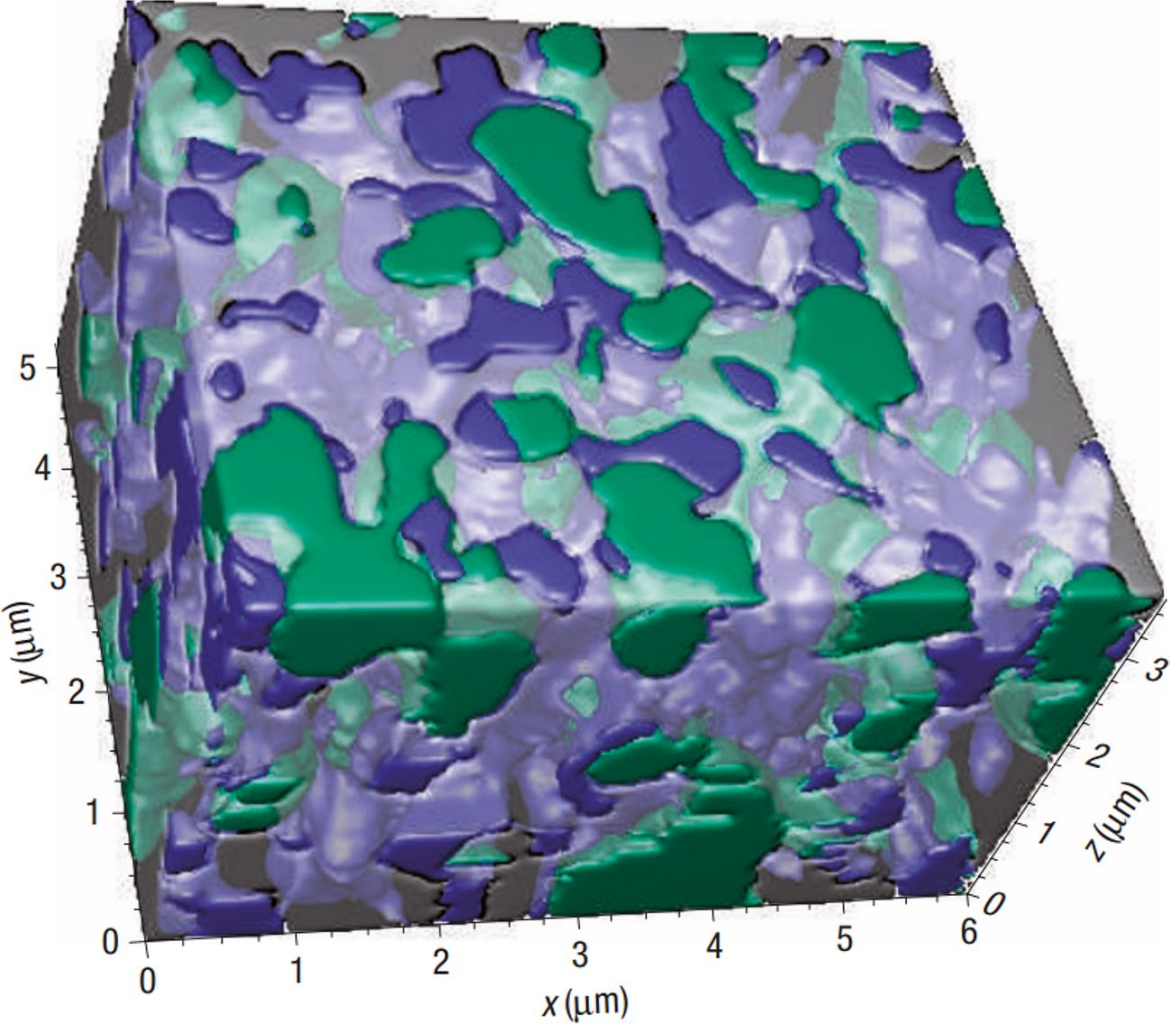
A view of the 3D reconstruction of a Ni-YSZ composite anode showing the Ni (green), YSZ (translucent/grey), and pore (blue) phases [75]

**Fig. 11:**
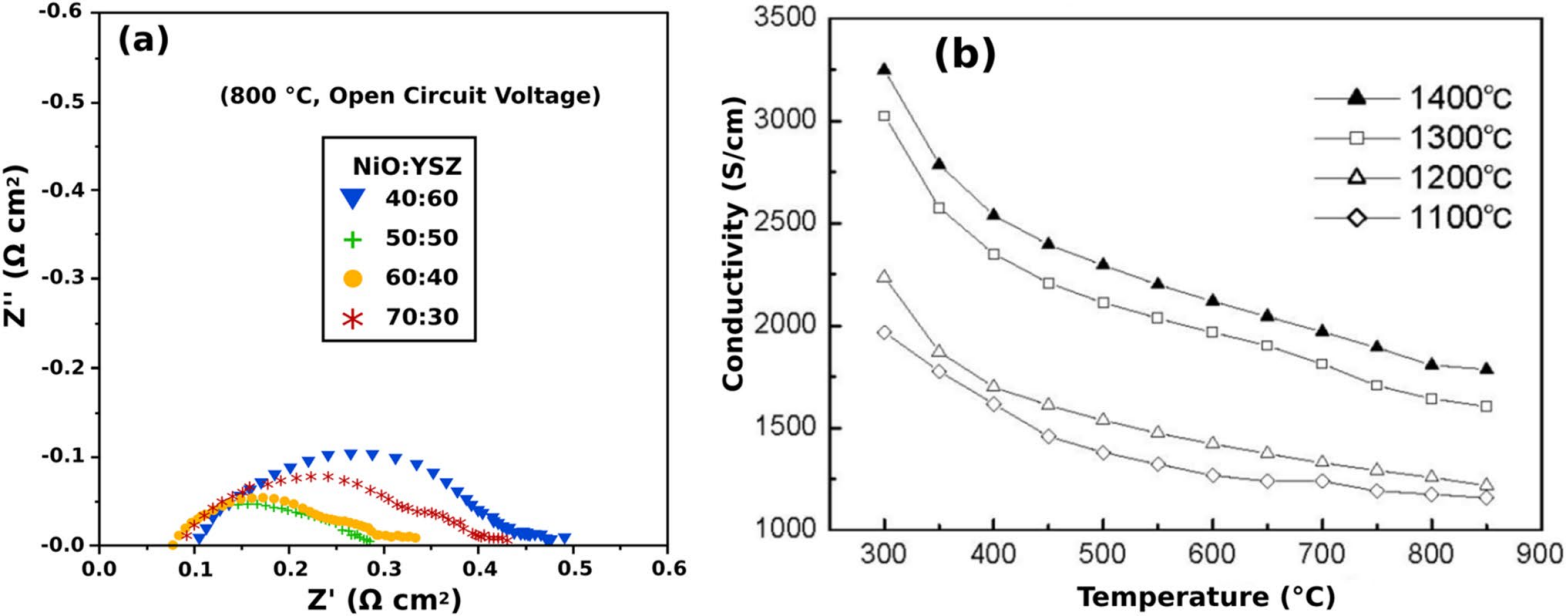
**a** Nyquist plots of EIS data for a Ni:YSZ composite anode measured at OCV in humidified hydrogen (reproduced from Wilson and Barnett (2008)) [62]. **b** Conductivity of a Ni-YSZ anode at different temperatures in H_2_ (reproduced from Kong et al. (2006)) [63]

Ni is a good candidate as an anode material for SOFCs owing to its low cost, high catalytic activity towards the HOR, and high conductivity [73]. However, poor adherence to ceramic electrolytes, structural instability, and no ionic conductivity prevent its use [61]. By creating a composite of Ni and a ceramic electrolyte (Ni-cermet), all three of these problems can be circumvented (Fig. 10) [74, 75]. An anode with 50 wt% NiO (reduces to Ni upon exposure to H_2_) and 50 wt% YSZ has high catalytic activity towards the HOR (0.2 Ω cm^2^ at 800 °C), high electrical conductivity (> 1000 S/cm at 800 °C), and the YSZ creates ion conducting networks (Fig. 11) [62, 63].

**Fig. 12:**
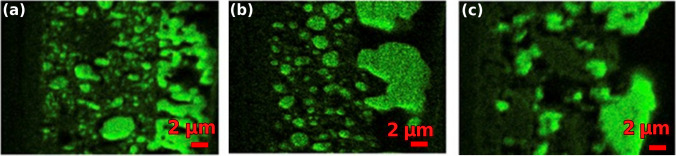
EDX-Mapping of a Ni anode after 2500 h (**a**), 15,000 h (**b**), and 20,000 h (**c**) of operation. The electrolyte is the dark area on the left, Ni is the green spots, and the anode surface is the dark area on the right [78]

At elevated temperatures (> 555 °C) and in the presence of moist hydrogen (> 1% H_2_O), Ni metal will be volatilized into Ni(OH)_2(g)_, evaporate inside the anode, and precipitate on the surface [76, 77]. Thus, long-term stability remains a problem for Ni-cermet anodes due to the depletion of nickel metal at the TPB and agglomeration of nickel particles reducing the active area (Fig. 12) [78].

#### Perovskite-type anodes

**Fig. 13:**
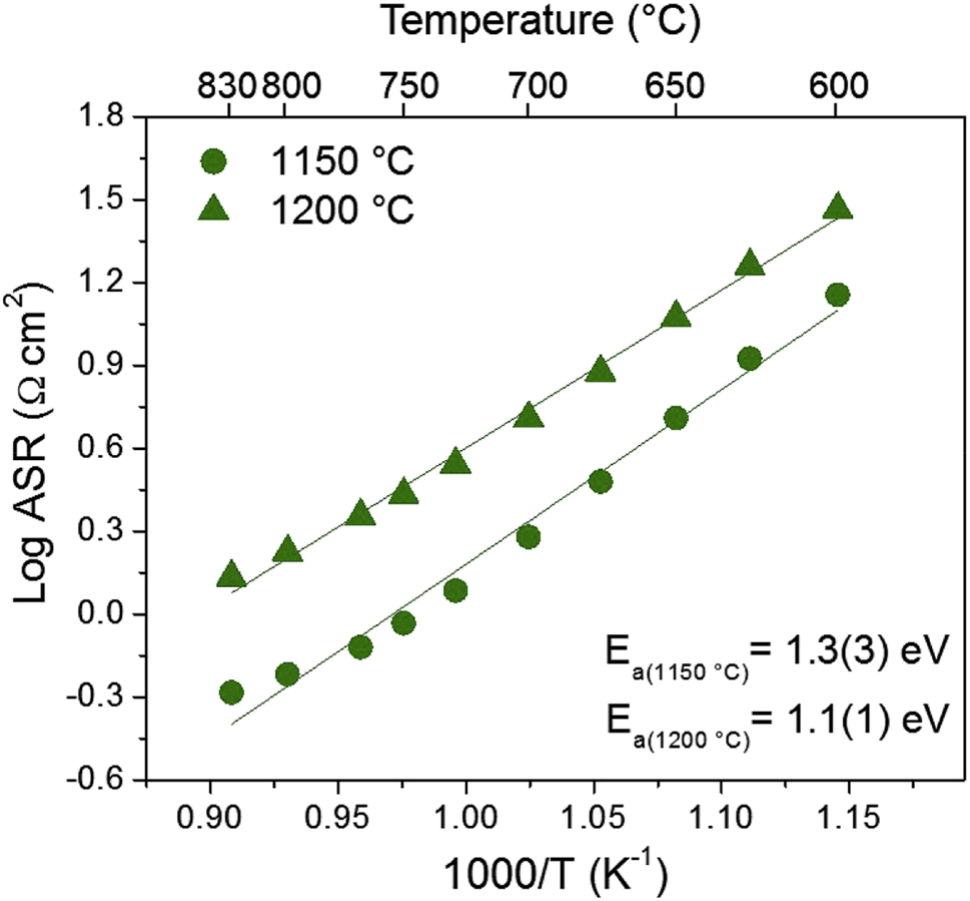
Arrhenius plots of ASR in humidified H_2_ for La_0.5_Sr_1.5_MnO_4+*δ*_ (L5S15M) electrodes sintered at 1150 and 1200 °C [81]

Several known perovskite-type oxides are unviable for SOFC anodes due to low catalytic activity towards the HOR, low conductivity, and instability in reducing environments, as they readily form oxide ion vacancies [79, 80]. The La_0.5_Sr_1.5_MnO_4+*δ*_ (L5S15M) Ruddleson-Popper was screened as a potential candidate as an SOFC cathode but its catalytic activity towards the HOR (0.61 Ω cm^2^ at 800 °C) is too low to replace the typical Ni metal anode (Fig. 13) [81].

**Fig. 14:**
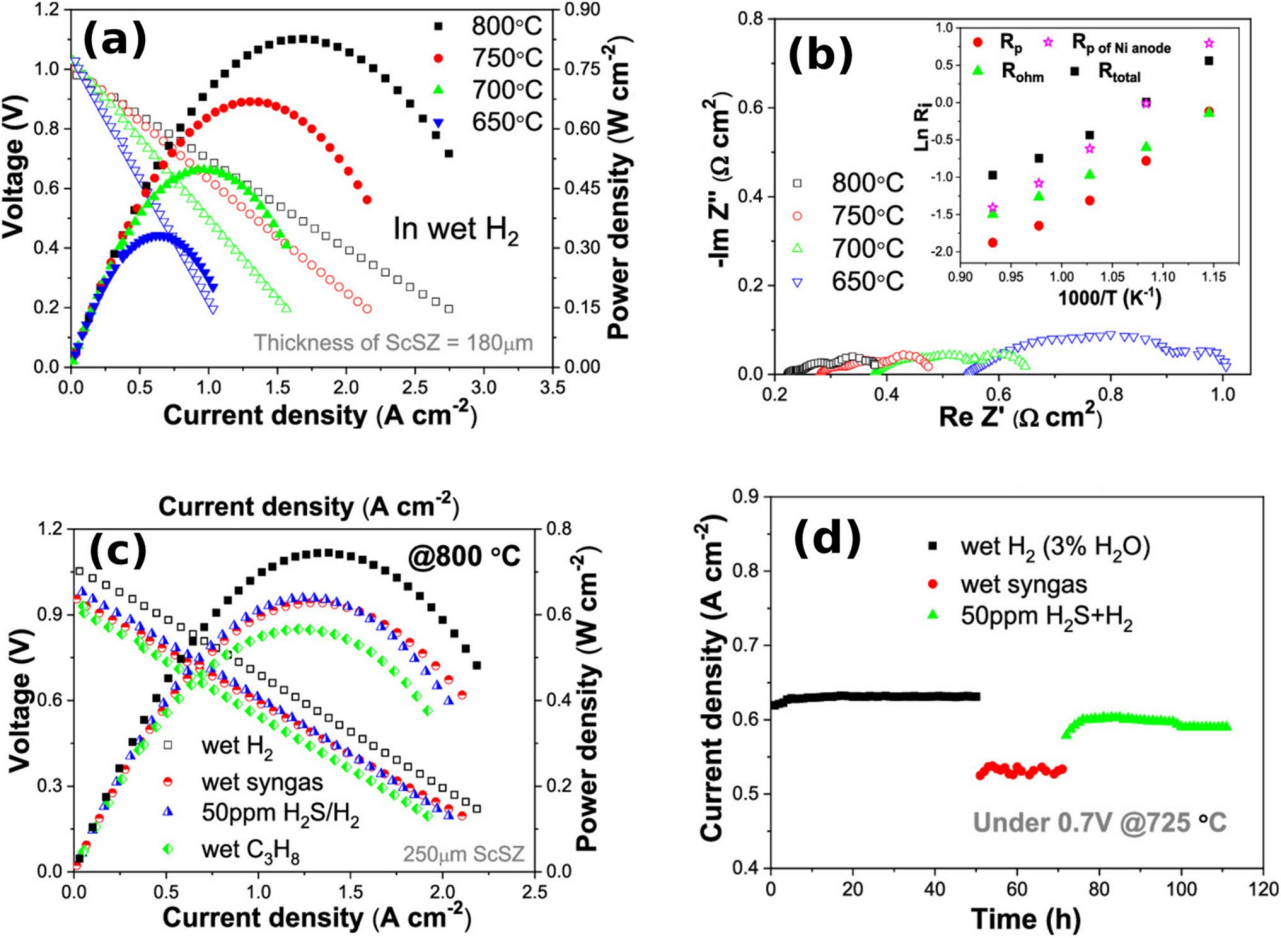
**a** I–V/P curves of a 180-μm ScSZ-supported single cell with the LSFNN anode in a temperature range of 600–800 °C; wet H2 (3% H2O) was fed to the anode as fuel, and ambient air was used as the oxidant. **b** EIS of the cell recorded under OCV; inset: Arrhenius plots of Rp and Rohm of the cell; for comparison, the Rp of a Ni-YSZ anode-based cell was also presented. **c** I–V/P curves of 250-μm ScSZ-supported single cells fed with various fuels at 800 °C. **d** Current density of a single cell in various fuels as a function of time at 725 °C under a constant voltage of 0.7 V [82]

The La_0.5_Sr_0.5_Fe_0.8_Ni_0.1_Nb_0.1_O_3−*δ*_ (LSCFNN) perovskite has been demonstrated in a full SOFC using a Sc-doped ZrO_2_ (ScSZ) electrolyte and a La_0.5_Sr_0.5_Fe_0.8_Cu_0.15_Nb_0.05_O_3−*δ*_ (LSFCN) cathode showing a low resistance (0.15 Ω cm^2^ at 800 °C, wet H_2_ fuel, air oxidant) and stability at 0.7 V for 50 h (Fig. 14) [82]. Symmetric cell testing should be done on La_0.5_Sr_0.5_Fe_0.8_Ni_0.1_Nb_0.1_O_3−*δ*_ to isolate for the anode performance.

The Sr_2_CrMoO_6−*δ*_ double-perovskite has higher electrical conductivity (92 S/cm at 800 °C in 5% H_2_) when compared to other Sr and Cr containing perovskite anodes such as La_0.75_Sr_0.25_Cr_0.5_Mn_0.5_O_3−*δ*_ (0.95 S/cm at 800 °C in 5% H_2_). This is attributed to the high concentration of Mo^6+^ and Cr^2+^ in the crystal structure which conduct electrons through the Mo^5+^/Mo^6+^ redox couple and Mo^6+^—Cr^2+^ double exchange interactions [68, 72]. It is also tolerant to H_2_S impurities in fuel which damage typical Ni-cermet anodes; however, the resistance towards the HOR is four times higher (0.71 Ω cm^2^ at 800 °C in H_2_) than typical Ni-cermet anodes limiting the total power capabilities [72].

#### Cu-cermet anodes

Cu-cermet anodes are typically used over Ni-cermet anodes in SOFCs utilizing hydrocarbons as fuel due to minimal coking at the anode surface [83]. However, Cu itself is not active for the HOR so ceria is added as the catalyst, copper provides the electronic conductivity (> 70 S/cm at 700 °C), and an ion conductor is added [83, 84]. When using H_2_ as fuel, Cu–CeO_2_–YSZ anodes show comparable activity towards the HOR (< 0.5 Ω cm^2^ at IT) as Ni-YSZ; when using CO as fuel, Cu-CeO_2_-YSZ shows ~ 3 × the performance when compared to Ni-YSZ [64, 85]. Thus, Cu–CeO_2_–YSZ is a good candidate for SOFCs operating in either hydrocarbons or hydrogen containing impurities. Other Cu-cermet anodes have shown copper migration and agglomeration so the long-term stability of these materials under SOFC conditions should be determined [86].

### Oxide ion electrolytes

**Fig. 15:**
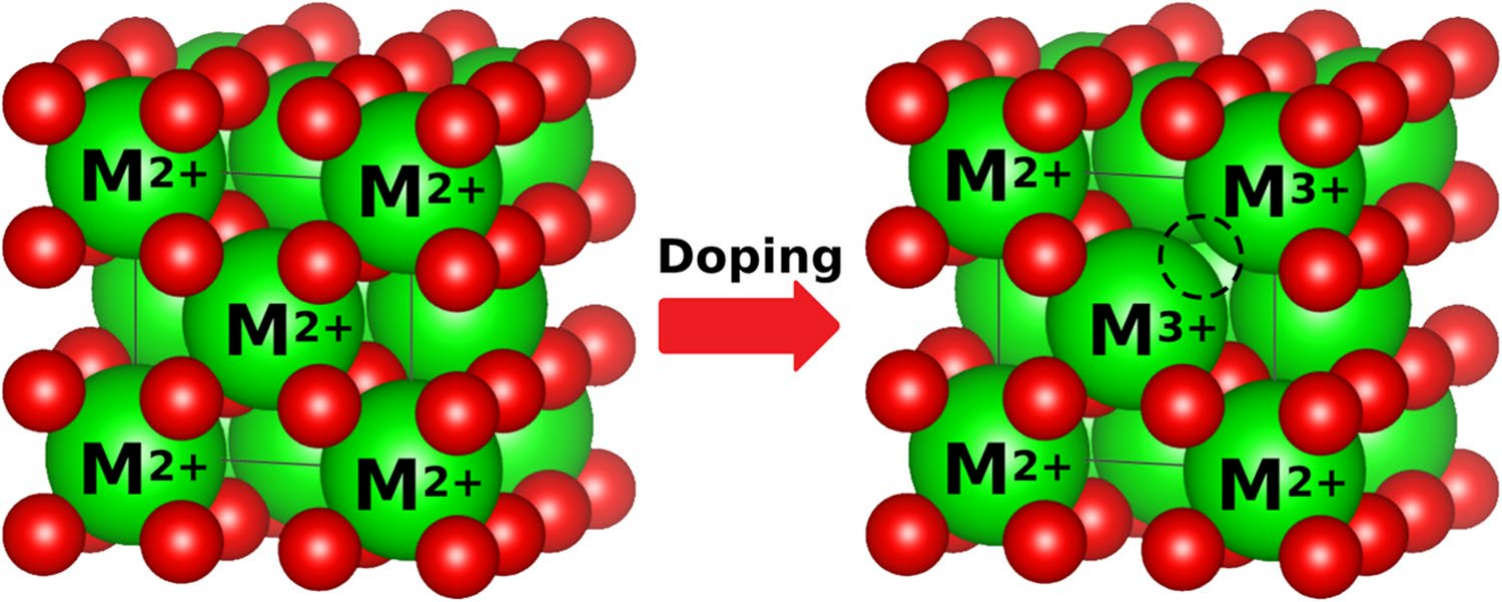
The mechanism for inducing oxygen vacancies

**Fig. 16:**
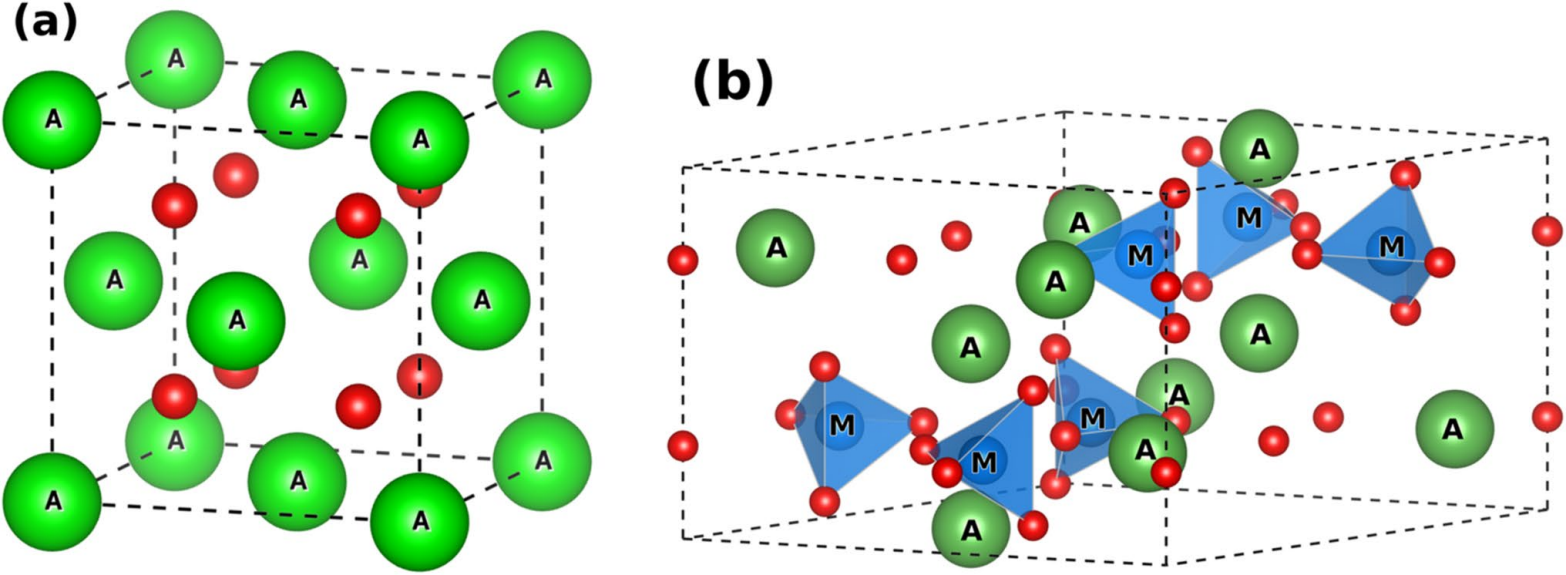
**a** The generalized crystal structure of an AO_2_ fluorite in the *Pa-3* space group (No. 205); the A-site at the 4*a*-Wyckoff positions; the oxygen site at the 4*b*-Wyckoff positions. **b** The generalized crystal structure of an A_10_(MO_4_)_6_O_2+*δ*_ appetite in the *P*6_3_*/m* space group (No. 176) (modified from ICSD 154068); the A-site at the 4*f* and 6* h*-Wyckoff positions; the M-site at the 6 m-Wyckoff positions; the oxygen site at the 2*a*, 6* h*, and 12*i*-Wyckoff positions [99]

Electrolyte materials for SOFCs are designed to meet the following criteria. (i) High oxide ion conductivity (> 0.1 S/cm at IT) to minimize the ohmic resistance with minimal electronic conductivity [87]. Ions are conducted via a vacancy hopping mechanism so a higher concentration of oxygen vacancies improves the ionic conductivity (Eq. 1 and Fig. 15) [88–90].

1 $$2{M}_{X}^{X}+{O}_{O}^{x}\to 2{M}_{M}^{\prime}++{V}_{O}^{\bullet \bullet }+\frac{1}{2}{O}_{2}$$

(ii) Structural stability under SOFC operating conditions (IT, oxidizing and reducing environment, dual-atmosphere, and polarization). It must form a dense structure to avoid gas diffusion through the electrolyte; and (iii) compatibility with the electrode and interconnect materials including chemical stability and a TEC match. Classes of materials for SOFC electrolytes that can meet the above criteria include fluorite-type, perovskite-type, and apatite-type electrolytes (Fig. 16 and Table 3) [91–98].

**Table 3:** Electrolyte materials for SOFC and their properties [91–98]

Material	Ionic conductivity (S/cm)	Thermal expansion coefficient (10^−6^/K)	Structure
Ce_0.8_Sm_0.2_O_1.95_	0.1 (800 °C) [91]	12.6 [92]	Fluorite
Ce_0.8_Gd_0.2_O_1.95_	0.086 (800 °C) [91]	12.2 [93]	Fluorite
(ZrO_2_)_0.92_(Y_2_O_3_)_0.08_	0.025 (800 °C) [94]	10.3 [93]	Fluorite
(ZrO_2_)_0.89_(Sc_2_O_3_)_0.1_(CeO_2_)_0.01_	0.04 (800 °C) [95]	10.5 [93]	Fluorite
La_0.8_Sr_0.2_Ga_0.8_Mg_0.2_O_3−*δ*_	0.15 (800 °C) [96]	11.6 [97]	Perovskite
La_9.5_(Ge_5.5_Al_0.5_O_24_)O_2_	0.16 (800 °C) [98]	8.9 [98]	Appetite

#### Fluorite-type electrolytes

**Fig. 17:**
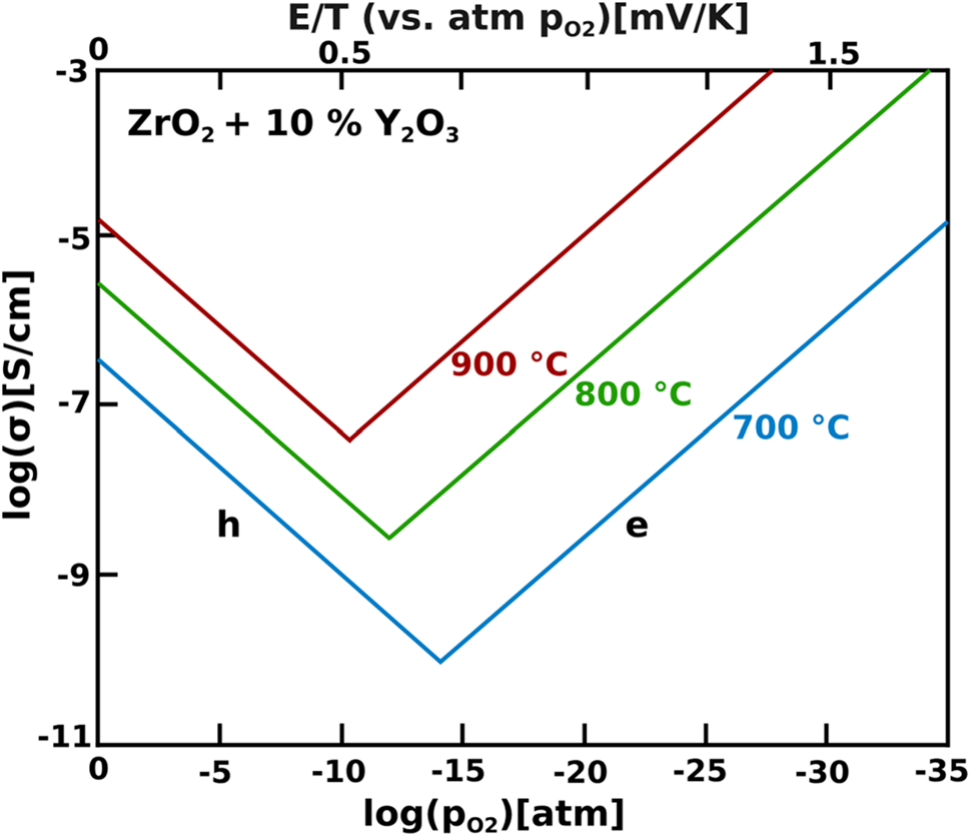
The dependence of the partial conductivities of electrons (e), and holes (h) in ZrO + 10% YzO_3_ due to the oxygen partial pressure (reproduced from Weppner (1977)) [102]

The fluorite structure is described by the chemical formula of AO_2_ where the cation sits at the 4-coordinate A-site located at the face and edge positions while oxygen sits at the interstitial sites (Fig. 16) [100]. ZrO_2_ is a good oxide-ion conductor when in the cubic fluorite phase, however, it only maintains this structure at temperatures above 2300 °C; upon quenching below 2300 °C, the cubic-fluorite structure decomposed into the poor oxide-ion conducting hexagonal structure [101, 102]. By doping Y_2_O_3_, the cubic ZrO_2_ structure is stabilized at low temperatures, and oxygen vacancies are introduced (Eq. 2 and Fig. 17) [103].

2 $${Y}_{2}{O}_{3}+Zr{O}_{2}\to 2 {Y}_{Zr}^{\prime}+{V}_{O}^{\bullet \bullet }+3 {O}_{O}^{x}$$

**Fig. 18:**
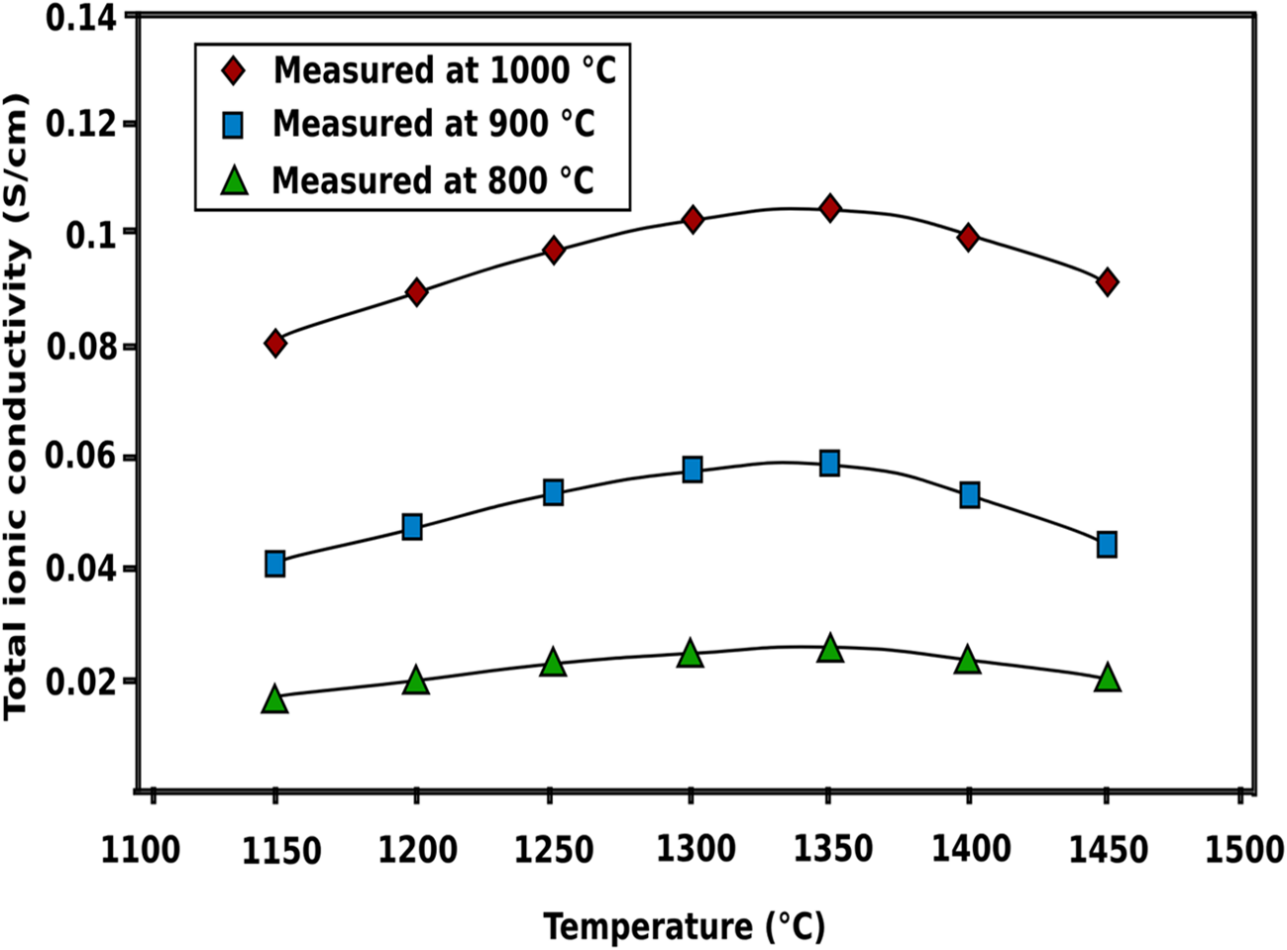
Total ionic conductivity as a function of sintering temperature for a YSZ electrolyte (reproduced from Chen et al. (2002)) [94]

Yttria stabilized zirconia (YSZ) has high ionic conductivity of 0.1 S/cm at 1000 °C but at ITs, the ionic conductivity is low (> 0.02 S/cm at 800 °C) making it impractical for IT-SOFCs (Fig. 18) [94, 96]. The cubic YSZ structure has also been shown to decompose during SOFC operating conditions which will lower the ionic conductivity with time [104].

**Fig. 19:**
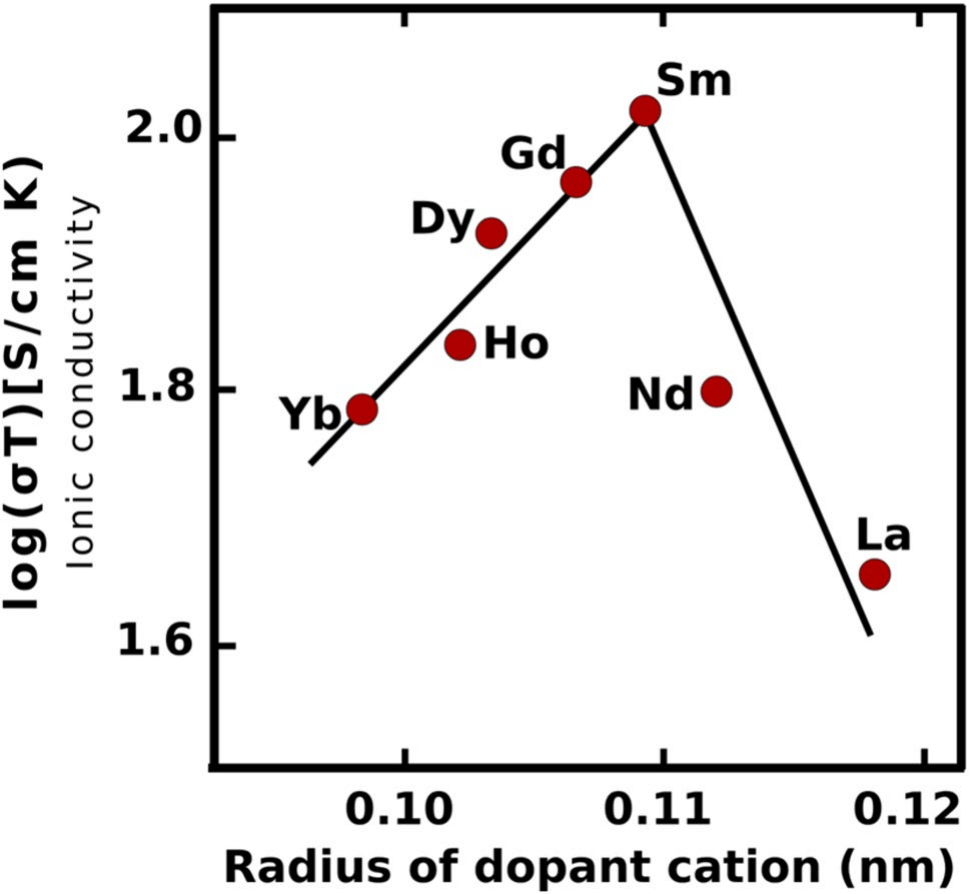
Dependence of ionic conductivity for (CeO_2_)_0.8_ (LnO_1.5_)_0.2_ (Ln = Dy, Gd, Ho, La, Nd, Sm, Y, and Yb) at 1073 K on radius of dopant cation (reproduced from Yahiro (1989)) [91]

Unlike ZrO_2_, CeO_2_ maintains the cubic fluorite structure at all temperatures but pure CeO_2_ is a mixed conductor and is reduced by H_2_ to give electronic conducting species making it unsuitable as an electrolyte [105]. Doping CeO_2_ with rare earth elements greatly raises the ionic conductivity and stabilizes CeO_2_ in reducing environments; samarium-doped ceria has an ionic conductivity that is an order of magnitude larger than YSZ and is the most stable in reducing atmospheres when compared to the other rare earth-doped CeO_2_ electrolytes (Fig. 19) [91].

#### Perovskite-type electrolytes

**Fig. 20:**
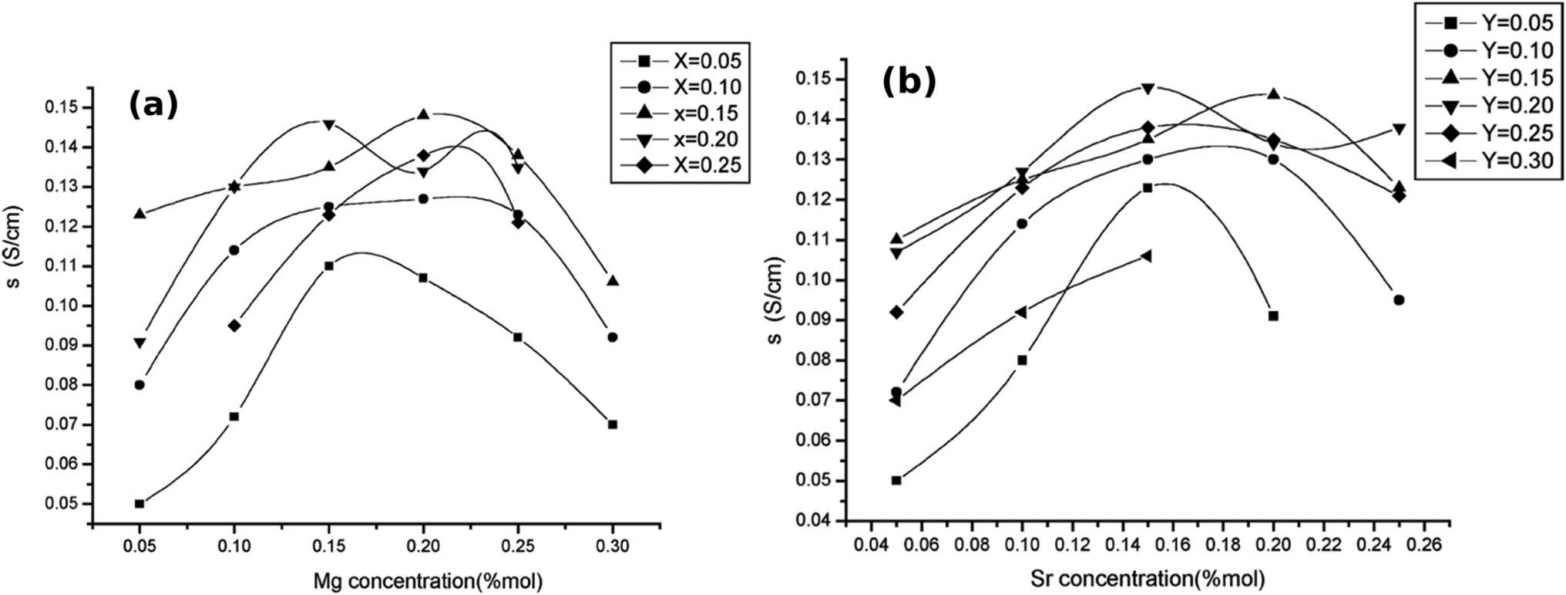
Ionic conductivity variation with concentration of (**a**) Mg and (**b**) Sr in La_1−*x*_Sr_*x*_Ga_1−*y*_Mg_*y*_O_3−*δ*_ at 800 °C [96]

Perovskite-type structures as electrolytes for SOFC are appealing due to their high ionic conductivity and oxygen-deficient structure allowing for oxide ion vacancy hopping [106]. La_1−*x*_Sr_*x*_Ga_1−*y*_Mg_*y*_O_3−*δ*_ (LSGM) type materials are very popular as SOFC electrolytes [107, 108]. LSGM has an ionic conductivity of ~ 0.1 S/cm at 800 °C (with negligible electronic conductivity) which is comparable to YSZ at 1000 °C (Fig. 20) [109].

**Fig. 21:**
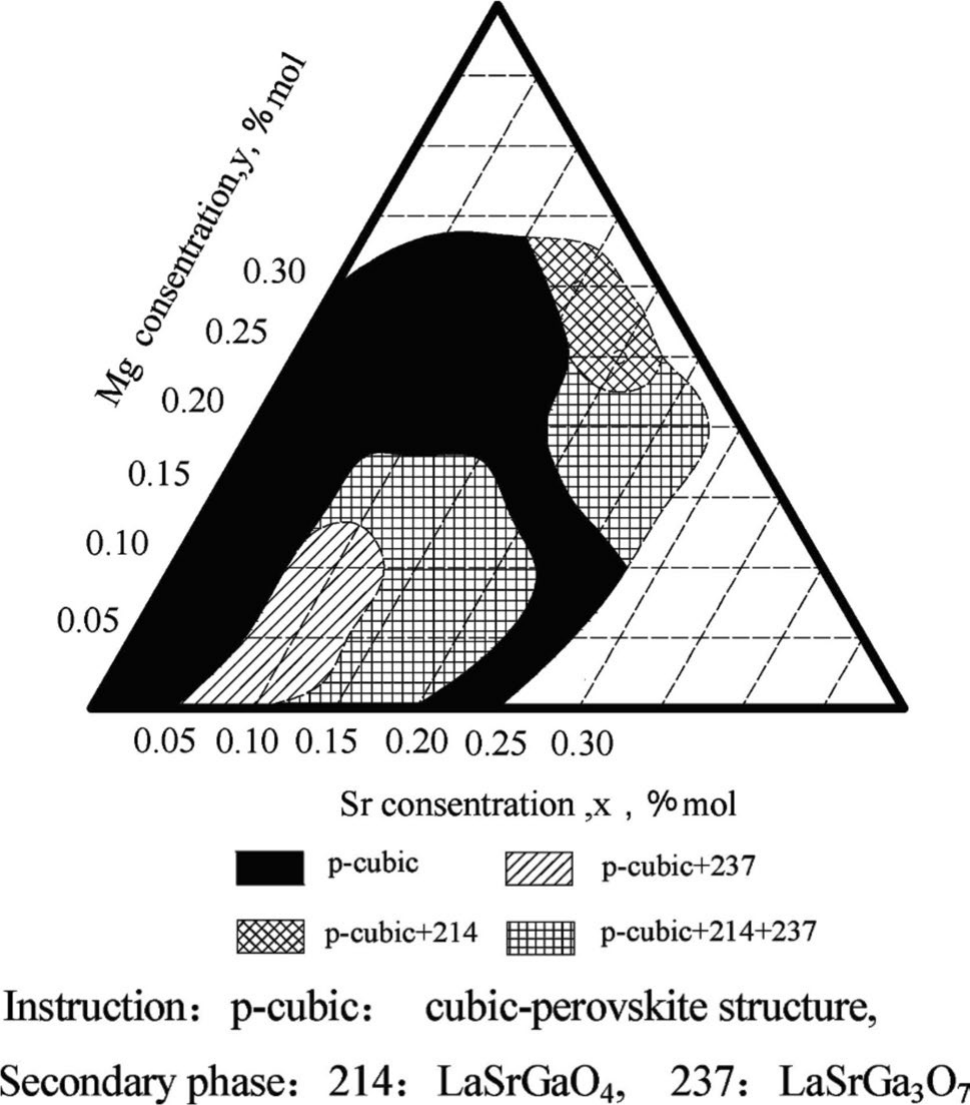
Phase region diagram of LSGM with various concentrations of Sr and Mg [96]

The lower operating temperature of LSGM makes it an appealing choice for IT-SOFC. However, synthesis of a single LSGM phase is difficult as the secondary phases of LaSrGaO_4_, LaSrGa_3_O_7_, La_4_Ga_2_O_9_, and/or LaGa_2_O_4_ form readily (Fig. 21) [96, 110]. It is also 2–5 × more expensive than other commercialized electrolytes (Fuelcellmaterials, USA). While the structural stability of LSGM is reported, its reactivity with the common nickel-cermet anodes is undesirable and limits its use as a SOFC electrolyte [111].

#### Apatite-type structure electrolytes

**Fig. 22:**
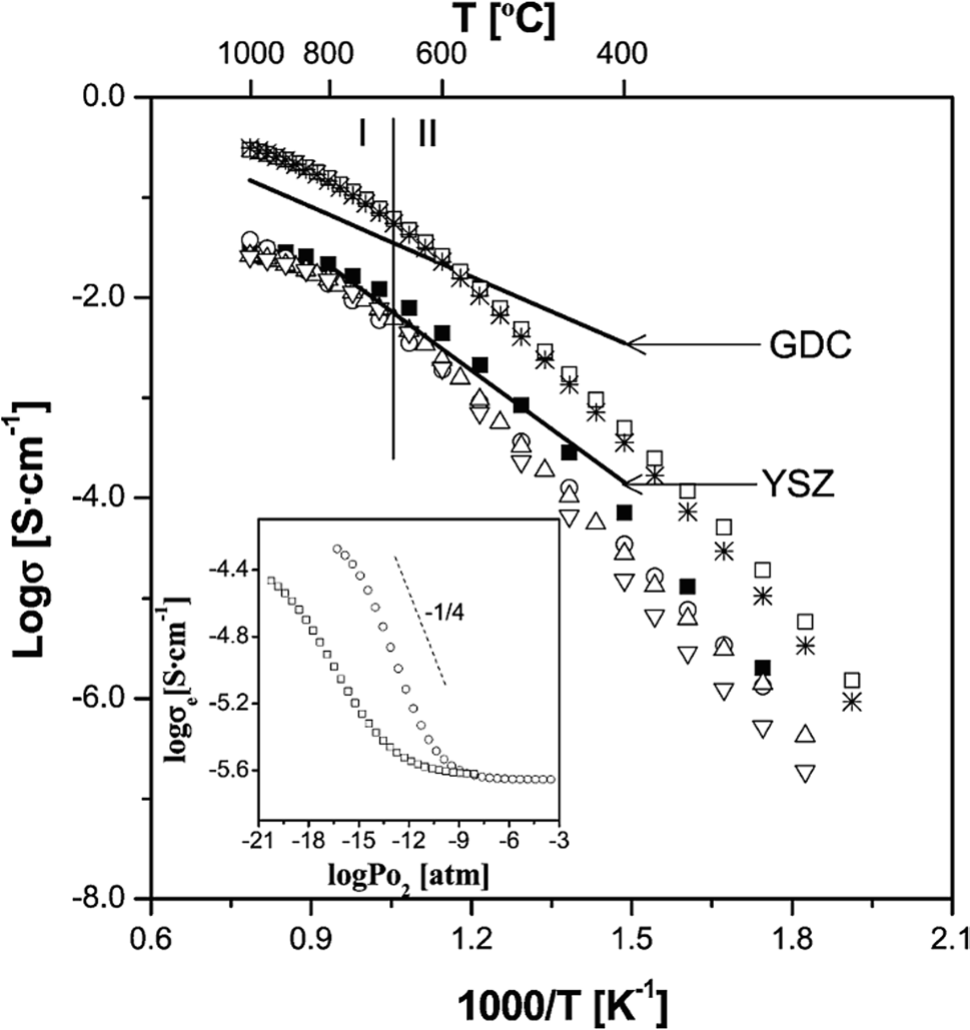
Arrhenius plot of the ionic conductivity for La_10−*x*_(Ge_5.5_Al_0.5_O_24_)O_2.75−1.5*x*_ (*x* = 0.5 (□/*), *x* = 0.4 (○), *x* = 0.33 (▵), *x* = 0.25 (▿). The inset shows the electronic conductivity for La_9.50_Al_0.5_ at 950 °C (□) and 1000 °C (○) from the ion-blocked technique [98]

The apatite structure is described by the general formula A_10_(MO_4_)_6_O_2+*δ*_ where the A-site is occupied by alkaline/rare earth metals and MO_4_ is a trivalent anionic group (Fig. 16) [112]. Unlike traditional oxide-ion conductors which conduct through oxygen vacancies, apatite-type materials conduct through interstitial oxygens [112, 113]. Fully stoichiometric apatite materials are poor oxide conductors; by creating excess oxygen or cation vacancies in the structure, the ionic conductivity can be increased by 2 orders of magnitude and 3 orders of magnitude respectively [114]. Thus, cation vacancies are shown to be the most important factor for the oxide conductivity in apatite-type materials. The La_10-*x*_(Ge_5.5_Al_0.5_O_24_)O_2.75−1.5×_ series has been shown to have comparable or higher ionic conductivity at ITs compared to YSZ with the *x* = 0.5 member showing the highest ionic conductivity of 0.16 S/cm at 800 °C (Fig. 22) [98]. However, prolonged heating at temperatures > 1350 °C results in GeO_2_ leaching out from the structure, lowering the ionic conductivity and limiting its compatibility to only electrodes that sinter/adhere at lower temperatures [115, 116].

### Interconnect materials

**Fig. 23:**
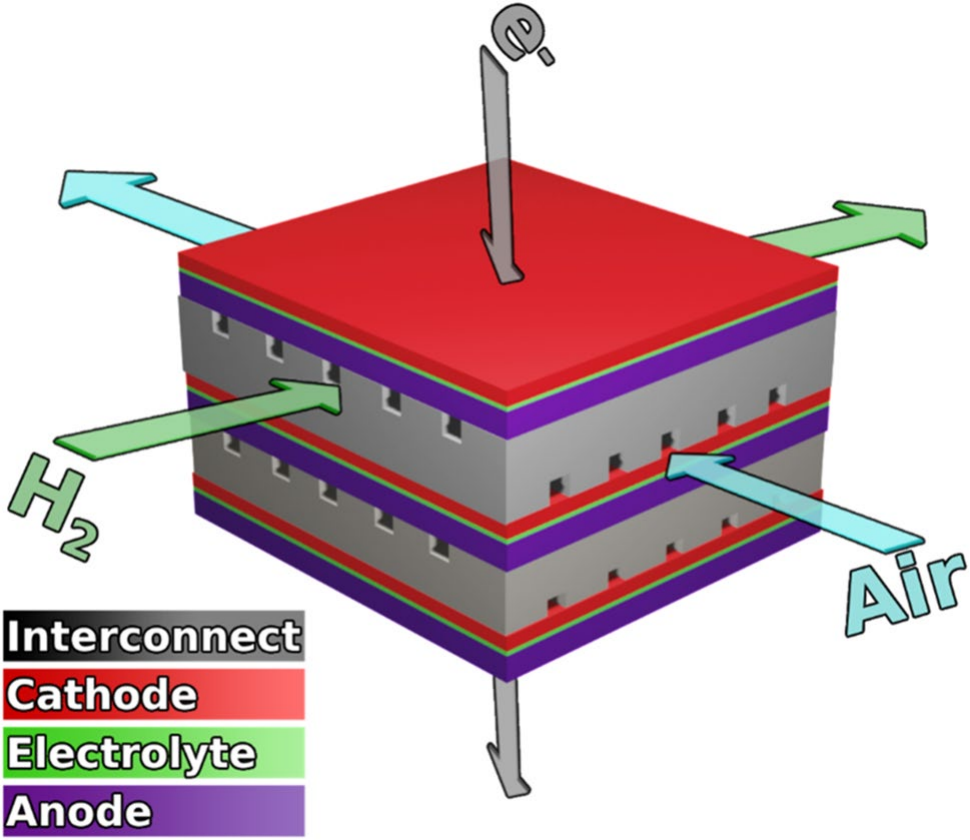
Schematic of an SOFC stack (reproduced from Heshmat and Cordova (2017)) [124]

As a single SOFC is only capable of producing a theoretical voltage of 1.23 V with maximum power occurring at ~ 0.55 V, several SOFCs must be connected in a “stack” which utilizes interconnects to join the anode of a single cell to the cathode of another cell (Fig. 23) [117, 118]. The interconnects function is to separate the cathode oxidizing environment of one cell from the anode reducing environment of another cell while allowing electrons to flow between the two cells [119]. They should meet the following criteria [120, 121]: (i) Under SOFC operating conditions, interconnects must be purely electronic conductors with a minimum electrical conductivity of 1 S/cm and ASR of 0.1 Ω cm^2^ to minimize the voltage losses due to the interconnect [122, 123]. (ii) Chemical and mechanical stability under SOFC operating conditions and in reducing/oxidizing environments. (iii) TEC match with the anode, cathode, and electrolyte. (iv) No permeability towards oxygen or hydrogen to avoid direct combination and lowering of the cell potential. Interconnect materials for SOFC that can meet these criteria are either metal or ceramic interconnects.

#### Ceramic interconnects

Lanthanum chromate (LaCrO_3_) and its doped alternatives are the most widely studied ceramic interconnect for SOFC owing to its moderate electrical conductivity under SOFC conditions, moderate stability in reducing and oxidizing environments, and compatibility with common SOFC materials [121, 125–127]. Undoped LaCrO_3_ conducts electrons through electron holes (h•) formed in the structure to compensate for cation vacancies (${V}_{La}^{{\prime}{\prime}{\prime}}+{V}_{Cr}^{{\prime}{\prime}{\prime}}$) according to the reaction [121]:

3 $$\frac{3}{2}{O}_{2}\to {V}_{La}^{{\prime}{\prime}{\prime}}+{V}_{Cr}^{{\prime}{\prime}{\prime}}+3 {O}_{O}^{x}+6 h\bullet$$

**Fig. 24:**
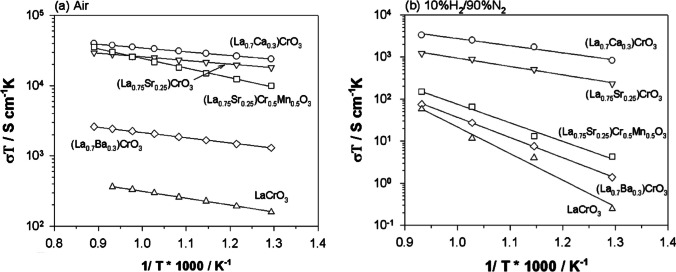
Activation energy plots of electrical conductivity for undoped LaCrO_3_, doped LaCrO_3_, and (La_0.75_Sr_0.25_)(Cr_0.5_Mn_0.5_)O_3_ perovskites in (**a**) air and (**b**) 10% H_2_/N_2_ [129]

Due to the dependence of the oxygen partial pressure on conductivity, the LaCrO_3_ conductivity at the anode side of the cell is low, rendering it unsuitable for use as an SOFC interconnect [128]. At 800 °C, LaCrO_3_ has a conductivity of 0.96 S/cm and 0.26 S/cm in air and 10%H_2_/90%N_2_ respectively (Fig. 24) [129]. However, this can be improved by doping alkaline earth elements (Ca, Sr, and Ba) at the A-site. Both La_0.75_Sr_0.25_CrO_3_ and La_0.7_Ca_0.3_CrO_3_ have a conductivity in oxidizing and reducing environments that is 2 orders of magnitude larger than that of LaCrO_3_ [129]. While Ba or Mg doping does increase the conductivity, the large size mismatch between La and Ba/Mg causes spinel phases to form which lower the conductivity due to electron-scattering [129, 130].

#### Metal interconnects

**Fig. 25:**
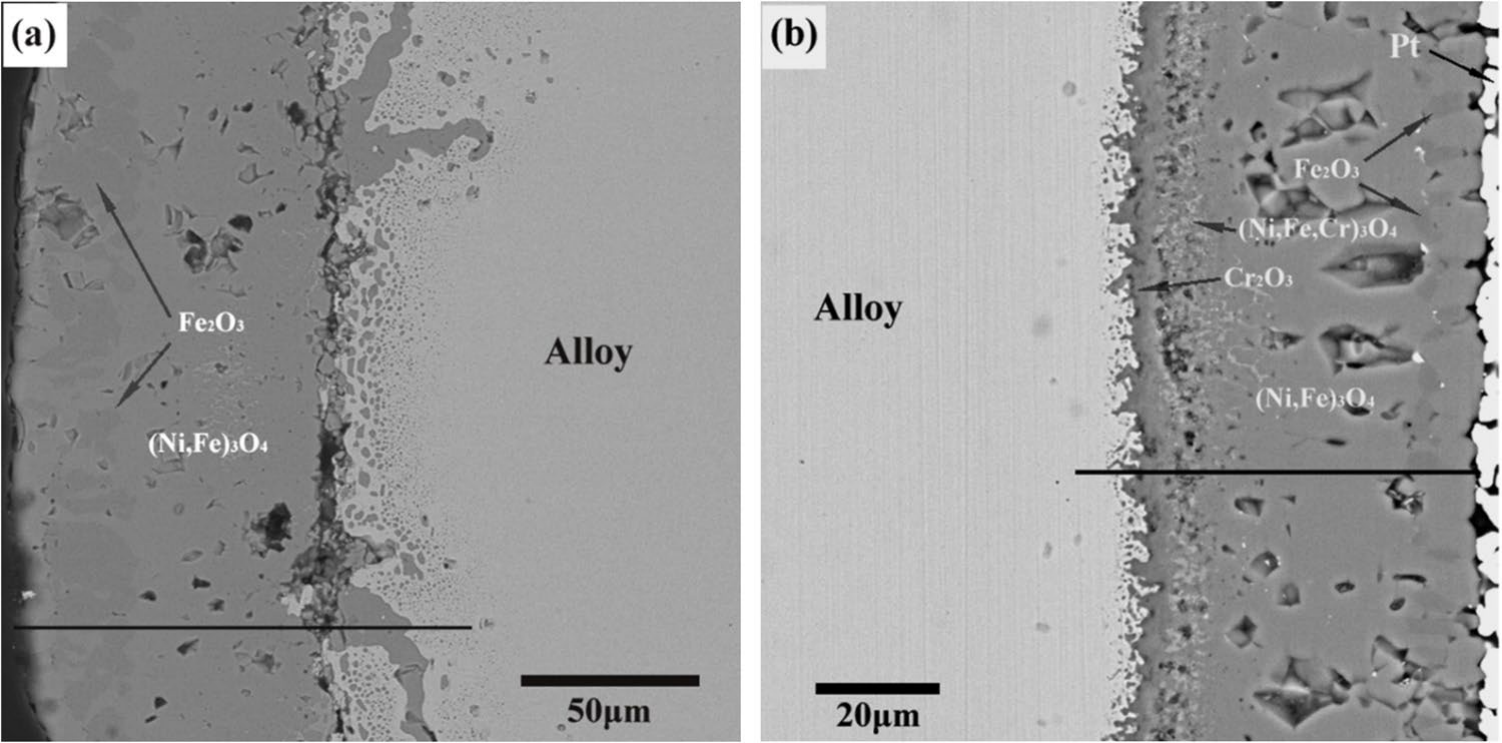
Cross-section SEM images of the oxide scales formed on (**a**) undoped Ni–Fe alloy and (**b**) 10 wt% Cr doped Ni–Fe alloy [134]

**Fig. 26:**
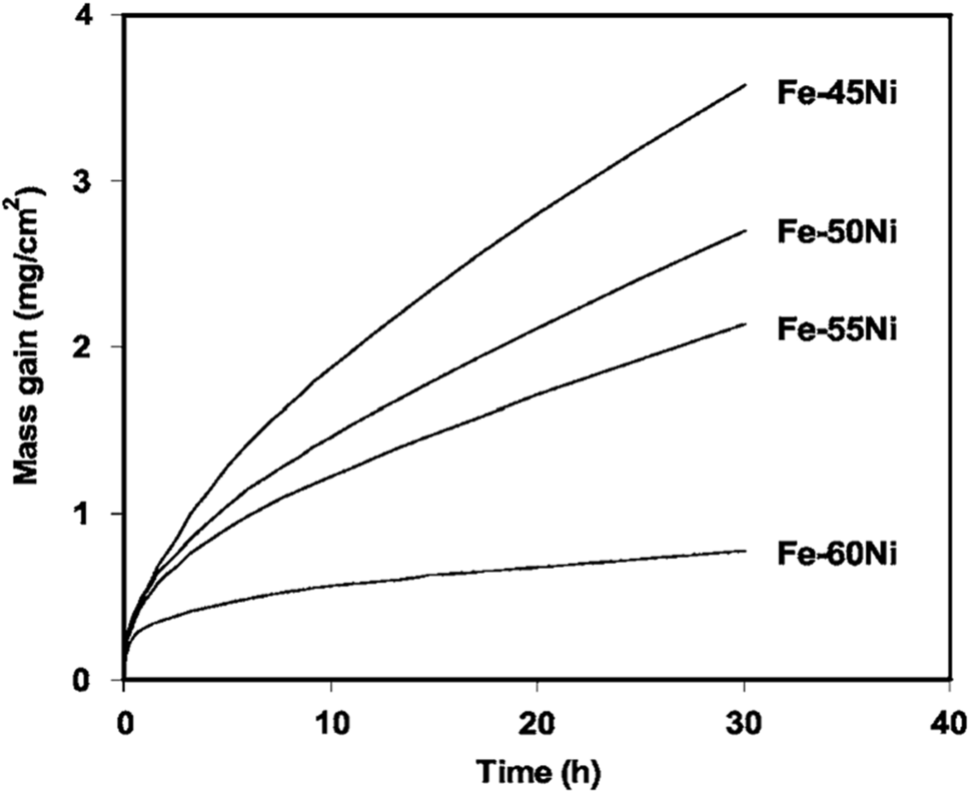
Mass gain (mg/cm^2^) due to oxide formation over time (h) for Fe–Ni alloys with various Ni wt% [135]

Under high temperature (> 800 °C) and SOFC operating conditions, metallic interconnects will oxidize losing mechanical strength and electronic conductivity with time [131]. However at ITs, metallic interconnects have superior strength and conductivity when compared to ceramic interconnects [119]. Cr/Al is added to Fe/Ni alloys to provide oxidative resistance by forming Cr_2_O_3_ and Al_2_O_3_ protective layers (Figs. 25 and 26) [132, 133].

**Fig. 27:**
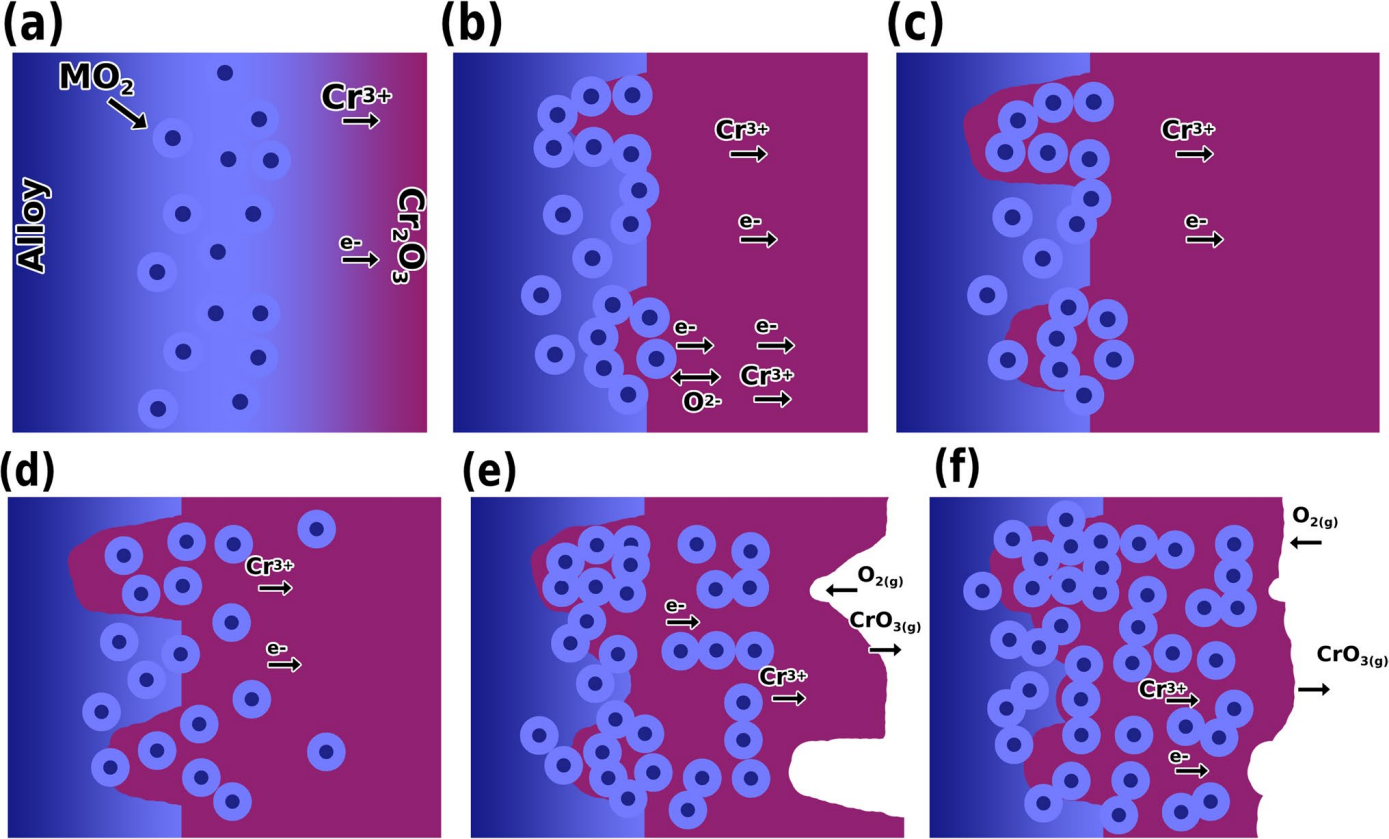
**a** A thin layer of Cr_2_O_3_ is formed via the outward diffusion of chromium, and the alloy-Cr_2_O_3_ interface becomes filled with agglomerates of MO_2_ particles (M = Fe, Ni, Th, etc.). **b** The MO_2_ agglomerates prevent the movement of chromium from the alloy and the Cr_2_O_3_ dissociates. Cr_2_O_3_ is formed beneath the MO_2_ agglomerates by the diffusion of oxygen through these particles. **c** The Cr_2_O_3_ formed beneath the M by the dissociation reaction becomes joined to the external scale upon continued growth of this type of Cr_2_O_3_ as well as growth of the external scale. **d** The inward growth mode that is caused by the agglomerated MO_2_ particles and the outward growth mode resulting from the diffusion of chromium in Cr_2_O_3_ produce a two-zoned Cr_2_O_3_ scale. **e** Portions of the outer zone are removed by the formation of CrO_3(g)_. **f** After long periods of oxidation virtually all of the outer zone is removed by the vaporization reaction. The MO_2_ particles in the Cr_2_O_3_ decrease the oxidation rate by decreasing the cross-sectional area of Cr_2_O_3_ available for the transport of chromium (reproduced from Whittle and Stringer (1980)) [136]

Reactive elements with a higher affinity towards oxygen than Cr will enhance the selective oxidation of Cr and suppress outward Cr diffusion reducing the growth rate of Cr_2_O_3_ (which leads to layer separation) caused when outward diffusing Cr reactions with inward diffusing oxygen; Al possesses the same properties but to a much lesser extent (Fig. 27) [136].

Al_2_O_3_ is not an electronic conductor like Cr_2_O_3_ so the Al content must be kept low to avoid forming a continuous insulating oxide layer [137, 138]. For Ni or Fe-based alloys, the addition of a maximum of 3 wt% Al is shown to lower the oxidation rate without the formation of an insulating Al_2_O_3_ layer [139]. A major problem with Cr-containing alloys is the Cr-poisoning of the cathode which increases the surface diffusion of oxygen and charge transfer resistance at the TPB [140, 141]. The mechanism for Cr-deposition at the cathode is best described using generalized nucleation deposition theory:

4 $${\mathrm{N}}_{\mathrm{a}}+{\mathrm{CrO}}_{3(\mathrm{g})}\to \mathrm{Cr}-\mathrm{N}-{\mathrm{O}}_{\left(\mathrm{nuclei}\right)}+{\mathrm{O}}_{2}$$

where N_a_ is the nucleation site (e.g., Mn, Sr, Ba, or Co) in the cathode or electrolyte, CrO_3(g)_ is vaporized Cr from the interconnect, and Cr–N-O_(nuclei)_ is the nuclei formed during the nucleation reaction [142, 143]. Cr–N-O_(nuclei)_ will further react with CrO_3(g)_ and deposit Cr in the form of Cr_2_O_3(s)_ and spinel-based (Cr,Mn)_3_O_4_, SrCrO_4_, BaCrO_4_, and CoCr_2_O_4_ for Mn, Sr, Ba, and Co respectively.

## Solid oxide fuel cell efficiency

### How is the Nernst potential of a solid oxide fuel cell influenced by gas pressure?

As SOFCs operate at elevated temperature and pressure (non-standard conditions), the Nernst equation is used to calculate the cell’s potential at various temperatures and pressures. The Nernst equation (Eq. 5) relates the cell’s potential (*E*) to its standard redox potential (1.229 V), the gas constant (*R*), temperature (*T*), the number of electrons transferred (*z*), Faraday’s constant (*F*), and the partial pressures of water (*P*_H2O_), O_2_ (*P*_O2_), and H_2_ (*P*_H2_).

5 $${E}_{\mathrm{nerst}}=1.229 V-\frac{RT}{zF}\mathrm{ln}\left(\frac{{P}_{{\mathrm{H}}_{2}\mathrm{O}}}{{\left({P}_{{\mathrm{O}}_{2}}\right)}^{1/2}{P}_{{\mathrm{H}}_{2}}}\right)$$

Using this formula, the effect of different gas environments on the Nernst potential can be predicted by keeping the temperature constant and assuming that *P*_O2_ or *P*_H2_ can change independently of *P*_H2O_ to isolate these three variables.

In case one, a cell using air (*P*_O2_ = 2.1 bar) as an oxygen source for the cathode is fitted with a pure oxygen tank (*P*_O2_ = 10 bar). This change is predicted to increase the Nernst potential by 30 mV; however, this small increase in potential may not justify the energy required to obtain pure oxygen [144]. In case two, the hydrogen supplied to the anode is hydrated (97% H_2_ + 3% H_2_O) to reduce the dual atmosphere effect [145, 146]. By hydrating the hydrogen, the *P*_H2_ decreases from 10 to 9.7 bar which decreases the Nernst potential by 1.1 mV. In this case, hydrating the hydrogen will improve the lifespan of the working SOFC with minimal voltage losses.

### What is the thermodynamic efficiency of a solid oxide fuel cell?

Energy conversion efficiency is described as the ratio between output energy over input energy. For an ideal solid oxide fuel cell, the input energy is the enthalpy of reaction for the water formation reaction $\left(\Delta {\rm H}_{f,{H}_{2}O}\right)$ while the output energy is the $\Delta {\rm H}_{f,{H}_{2}O}$ subtracted by the energy losses $\left(\Delta {S}_{{H}_{f,{H}_{2}O}}\right)$ per unit temperature (*T*) (Eq. (6) [147]. These energy losses include activation, ohmic, and concentration losses [148, 149]. While Eq. 6 shows that the efficiency decreases with increasing temperature, in working SOFCs, this is not the case. The activation, ohmic, and heat transfer losses become significant in SOFCs operating at temperatures below 600 °C which causes Δ*S* to become much larger than *T*, greatly lowering the efficiency of the cell at low temperatures [149–151].

6 $${\varepsilon }_{\mathrm{thermo}}=\frac{\Delta H-T(\Delta {S}_{\mathrm{ohm}}+\Delta {S}_{\mathrm{act}}+\Delta {S}_{h})}{\Delta H}=\frac{\Delta G}{\Delta H}$$

### What is the fuel efficiency of a solid oxide fuel cell?

The fuel utilization of a theoretical SOFC is the ratio between the moles of electrons produced per second (*ν*_charge_) and the moles of fuel supplied per second (*ν*_fuel_) (Eq. 7).

7 $${\varepsilon }_{\mathrm{fuel}}=\frac{{\nu }_{\mathrm{charge}}}{{\nu }_{\mathrm{fuel}}}$$

The *ν*_charge_ of a SOFC is the current density (i), divided by the charge produced by the reaction (*zF*) where *z* is the number of electrons produced by the reaction and *F* is Faraday’s constant. The *ν*_fuel_ is the volumetric flow rate (*Q*) converted to molar flow rate using the density (*ρ*) and molar mass (*M*) of the fuel. Thus, the fuel utilization for a fuel cell is given by Eq. 8.

8 $${\varepsilon }_{\mathrm{fuel}}=\frac{iM}{QzF\rho }$$

**Fig. 28:**
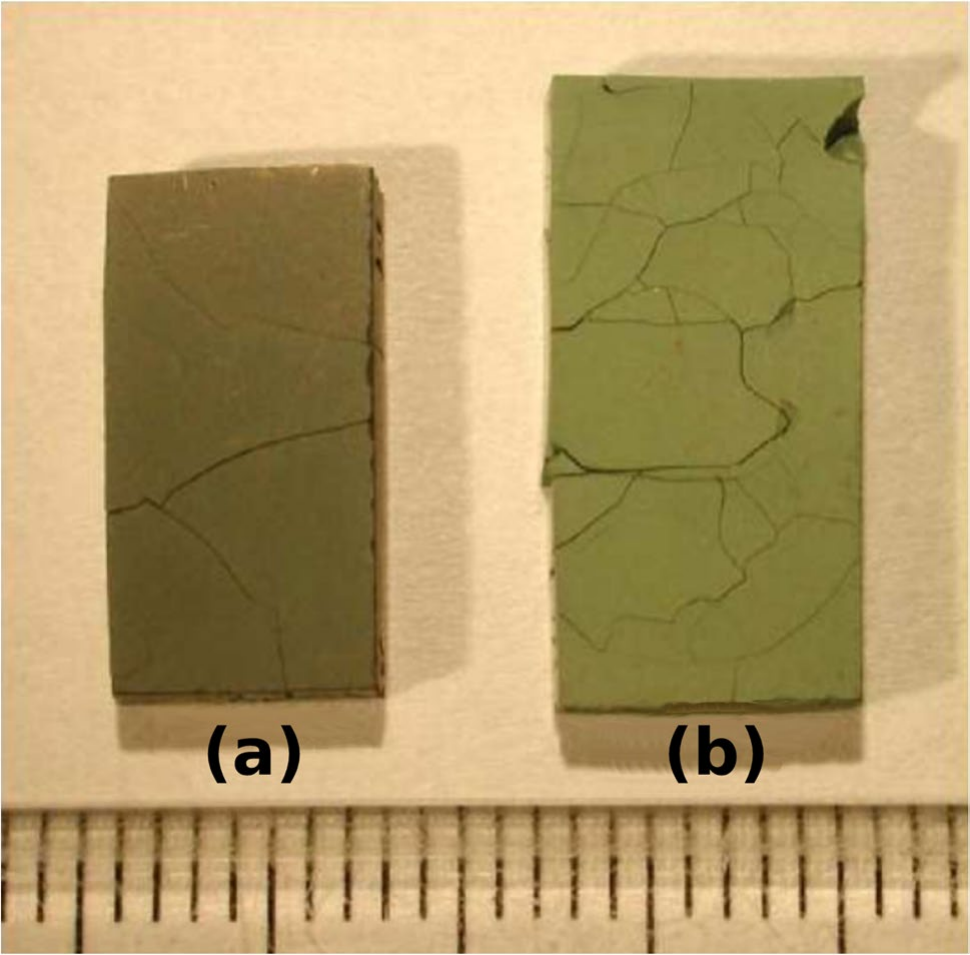
Images of a Ni-YSZ functional layer re-oxidized at 600 °C (**a**) and 750 °C (**b**) (graduations in mm) [159]

At maximum fuel efficiency, the hydrogen pressure at the anode will drop to nearly 0. The anode is at risk for reoxidation if the oxygen partial pressure (due to steam) is larger than the hydrogen partial pressure [152, 153]. During reoxidation, the anode will expand and crack (Fig. 28); this behavior is heavily reported for Ni-anodes [154–157]. Thus, fuel efficiency is kept at ~ 75% to maintain the reducing environment at the anode [158].

Hydrogen recovery is often used in SOFCs due to the simplicity of separating steam from gases. One method is to create a *dead-end loop* at the anode such that the outlet is sealed so all of the supplied hydrogen is utilized [160]. These systems must be pressure controlled to maintain a higher hydrogen pressure compared to steam to avoid anode reoxidation. While keeping the anode under constant pressure, steam is expelled through a condenser while the total pressure is maintained by flowing hydrogen into the system [161]. A second method is to recirculate the anode off gas back into the fuel stream using a pump/blower and a condenser to remove the steam [162, 163]. The dead-end loop consumes no energy but is unsuitable for high-power SOFCs with rapid fuel consumption; pumps can recirculate at high pressure but oil-lubrication of mechanical parts can contaminate the fuel being recirculated and oil-free pumps face durability problems; and blowers can recirculate large volumes of gas but at low pressure [163, 164].

### What is the voltaic efficiency of a solid oxide fuel cell?

The Nernst potential (Eq. 5) of a SOFC is the open-circuit voltage (OCV) assuming complete reversibility of all electrochemical processes within the cell. When drawing current from the SOFC, the cell potential will drop due to overpotential losses [149]. The voltaic efficiency is the ratio between the output cell potential and the Nernst potential (Eq. 9) [165].

9 $${\varepsilon }_{\mathrm{volt}}=\frac{{E}_{\mathrm{nernst}}-{\eta }_{\mathrm{act}}-{\eta }_{\mathrm{ohm}}-{\eta }_{\mathrm{conc}}}{{E}_{\mathrm{nernst}}}$$

**Fig. 29:**
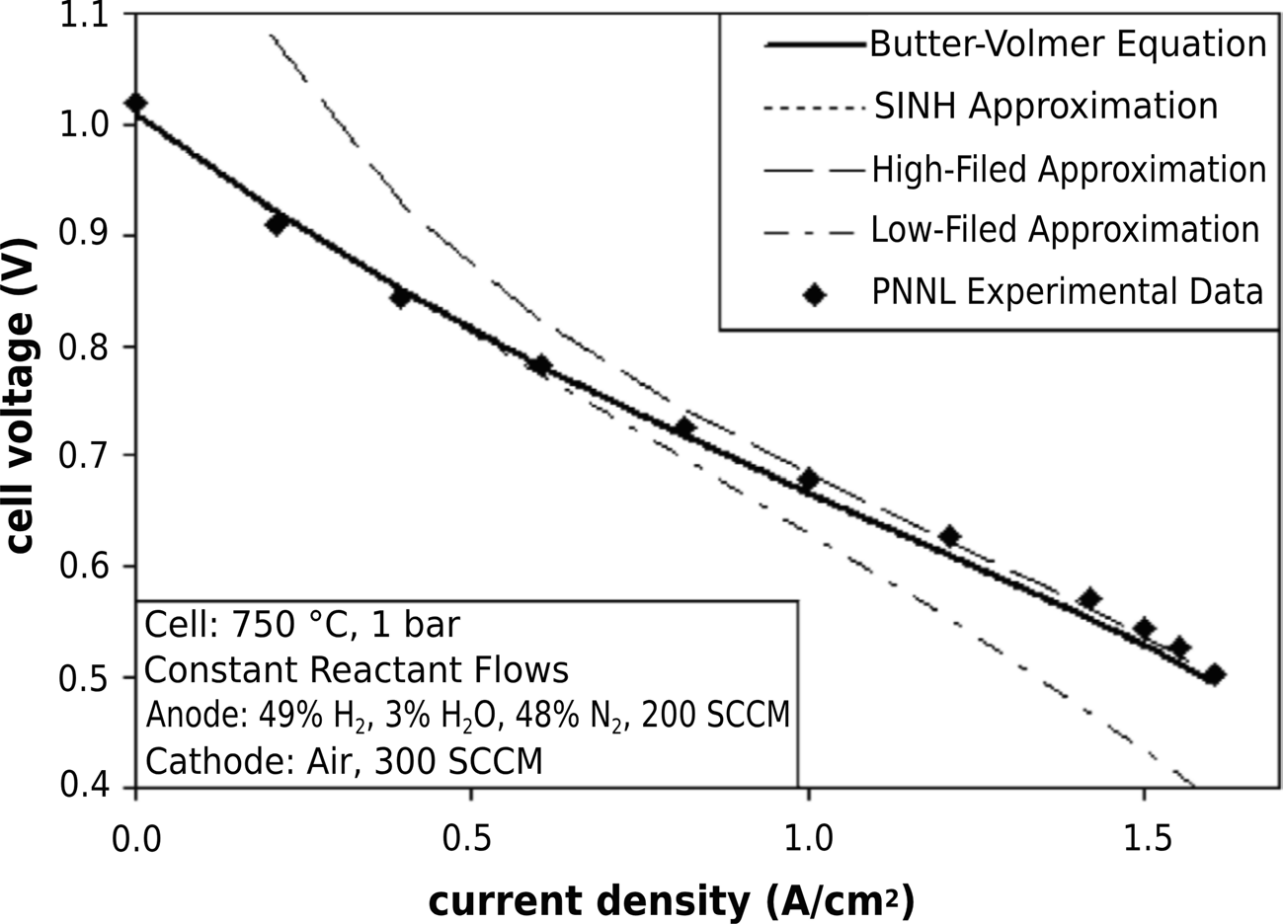
Comparison of experimental voltage–current data and model-based curves using different activation loss equations [168]

The activation overpotential is the potential required to overcome the activation barrier of the cell; this is related to the charge transfer and gas adsorption/desorption on the electrode surface [166]. For purely electronic conducting electrodes where the TPB exists only at the electrode–electrolyte interface, the activation overpotential (*η*_act_) is quantified by Eq. 10 where *i* is the operating current density of the cell and *i*_*o,a*_ and *i*_*o,c*_ are the exchange current densities of the anode and cathode respectively [167, 168]. The hyperbolic sine approximation was used over the Butler-Volmer equation due to its computational simplicity with nearly identical results (Fig. 29).

10 $${\eta }_{\mathrm{act}}=\frac{RT}{F}\left({\mathrm{sinh}}^{-1}\left(\frac{i}{2{i}_{o,a}}\right)+{\mathrm{sinh}}^{-1}\left(\frac{i}{2{i}_{0,c}}\right)\right)$$

The ohmic overpotential (*η*_ohm_) is the potential required to overcome the internal resistance of the cell [169]. It is quantified by Ohm’s law where *i* is the operating current density of the cell, *R*_*p*_ is polarization the resistance of the electrodes, and *R*_*s*_ is the series resistance at the electrolyte (Eq. 11) [149].

11 $${\eta }_{\mathrm{ohm}}=i\left({R}_{s}+{R}_{P}\right)$$

The concentration overpotential (*η*_conc_) is the potential lost due to the gas concentration gradient between the inlet and the TPB of the porous electrodes [170]. It results from fast electrochemical reactions coupled with slow gas diffusion within the porous electrodes most often occurring at high current [166]. *η*_conc_ is quantified using Eq. 12 where *P* is the pressure of the gas at the inlet/electrode surface and *P*_TPB_ is the pressure of the gas at the TPB [171].

12 $${\eta }_{\mathrm{conc}}=-\frac{RT}{2F}\mathrm{ln}\left(\frac{{P}_{{\mathrm{H}}_{2\left(\mathrm{TPB}\right)}}{\left({P}_{{\mathrm{O}}_{2\left(\mathrm{TPB}\right)}}\right)}^\frac{1}{2}{P}_{{\mathrm{H}}_{2}\mathrm{O}}}{{P}_{{\mathrm{H}}_{2}}{\left({P}_{{\mathrm{O}}_{2}}\right)}^\frac{1}{2}{P}_{{\mathrm{H}}_{2}\mathrm{O}\left(\mathrm{TPB}\right)}}\right)$$

For purely electronic conducting electrodes where the TPB exists only at the electrode–electrolyte interface, *P*_H2(TPB)_ and *P*_H2O(TPB)_ (atm) are related to the total H_2_ (*P*_H2_) and H_2_O (*P*_H2O_) (atm) pressure respectively, the current density of the cell (*i* (A/cm^2^)), the length of the anode diffusion path (*L*_anode_ (cm)) which is assumed to be the thickness of the anode, and the effective diffusion of the anode (*D*_eff,anode_ (cm^2^/s)) (Eqs. 13 and 14); *P*_O2(TPB)_ is related to total pressure at the cathode (*P*_c_ (atm)), the total pressure of O_2_ (*P*_O2_ (atm)), the length of the cathode diffusion path (*L*_cathode_ (cm)) which is assumed to be the thickness of the cathode, and the effective diffusion of the cathode (*D*_eff,cathode_ (cm^2^/s)) (Eq. 15) [149, 167, 172].

13 $${P}_{{\mathrm{H}}_{2}(\mathrm{TPB})}={P}_{{\mathrm{H}}_{2}}-\frac{iRT{L}_{\mathrm{anode}}}{2F{D}_{\mathrm{eff},\text{ anode}}}$$

14 $${P}_{{\mathrm{H}}_{2}\mathrm{O}(\mathrm{TPB})}={P}_{{\mathrm{H}}_{2}\mathrm{O}}+\frac{iRT{L}_{\mathrm{anode}}}{2F{D}_{\mathrm{eff},\text{ anode}}}$$

15 $${P}_{{\mathrm{O}}_{2}(\mathrm{TPB})}={P}_{c}-\left({P}_{c}-{P}_{{\mathrm{O}}_{2}}\right)\mathrm{exp}\left(\frac{iRT{L}_{\mathrm{cathode}}}{4F{D}_{\mathrm{eff},\text{ cathode}}}\right)$$

The effective diffusion of binary gases through a porous electrode (*D*_eff_ (cm^2^/s)) in SOFCs is modeled using binary diffusion (*D*_*ij*_ (cm^2^/s)) involving the interaction of gas species *i* and *j* where *T* is temperature (K), *P* is the total pressure (atm), *V* is the Fuller diffusion volume (cm^3^/mol) (7.07 for H_2_, 17.9 for N_2_, 16.6 for O_2_, and 12.7 for H_2_O), *M* is the molar mass of the gas (g/mol), and 10^−3^ is an arbitrary constant from the least squares fitting; and the Knudsen diffusion (*D*_*i*_^*k*^ (cm^2^/s)) involving the interaction of gas species *i* with the walls of the porous structure where *d*_*p*_ is the mean pore diameter (cm) ((Eq. 16) [173–176].

16 $$\frac{1}{{D}_{\mathrm{eff}}}={\left(\frac{{10}^{-3}{T}^{1.75}}{P{\left({V}_{i}^\frac{1}{3}+{V}_{j}^\frac{1}{3}\right)}^{2}}\sqrt{\frac{1}{{M}_{i}}+\frac{1}{{M}_{j}}}\right)}^{-1}+{\left(\frac{{d}_{p}}{3}\sqrt{\frac{8RT}{\pi {M}_{i}}}\right)}^{-1}$$

Combining Eqs. 12–16 gives the formula for concentration overpotential for SOFCs using experimentally measurable variables (Eq. 17).

17 $${\eta }_{\mathrm{conc}}=-\frac{RT}{2F}\mathrm{ln}\frac{\begin{array}{c}\left({P}_{{\mathrm{H}}_{2}}-\frac{iRT{L}_{\mathrm{anode}}}{2F}\left({\left(\frac{{10}^{-3}{T}^{1.75}}{{P}_{A}{\left({V}_{{\mathrm{H}}_{2}}^\frac{1}{3}+{V}_{{\mathrm{H}}_{2}\mathrm{O}}^\frac{1}{3}\right)}^{2}}\sqrt{\frac{1}{{M}_{{\mathrm{H}}_{2}}}+\frac{1}{{M}_{{\mathrm{H}}_{2}\mathrm{O}}}}\right)}^{-1}+{\left(\frac{{d}_{p,A}}{3}\sqrt{\frac{8RT}{\pi {M}_{{\mathrm{H}}_{2}}}}\right)}^{-1}\right)\right)\times \\ {\left({{P}_{c}-({P}_{c}-P}_{{\mathrm{O}}_{2}})\mathrm{exp}\left(\frac{iRT{L}_{\mathrm{cathode}}}{4F}\left({\left(\frac{{10}^{-3}{T}^{1.75}}{{P}_{C}{\left({V}_{{\mathrm{O}}_{2}}^\frac{1}{3}+{V}_{{\mathrm{N}}_{2}}^\frac{1}{3}\right)}^{2}}\sqrt{\frac{1}{{M}_{{\mathrm{O}}_{2}}}+\frac{1}{{M}_{{\mathrm{N}}_{2}}}}\right)}^{-1}+{\left(\frac{{d}_{p,C}}{3}\sqrt{\frac{8RT}{\pi {M}_{{\mathrm{O}}_{2}}}}\right)}^{-1}\right)\right)\right)}^\frac{1}{2}{P}_{{\mathrm{H}}_{2}\mathrm{O}}\end{array}}{{P}_{{\mathrm{H}}_{2}}{\left({P}_{{\mathrm{O}}_{2}}\right)}^\frac{1}{2}\left({P}_{{\mathrm{H}}_{2}\mathrm{O}}+\frac{iRT{L}_{\mathrm{anode}}}{2F}\left({\left(\frac{{10}^{-3}{T}^{1.75}}{{P}_{A}{\left({V}_{{\mathrm{H}}_{2}\mathrm{O}}^\frac{1}{3}+{V}_{{\mathrm{H}}_{2}}^\frac{1}{3}\right)}^{2}}\sqrt{\frac{1}{{M}_{{\mathrm{H}}_{2}\mathrm{O}}}+\frac{1}{{M}_{{\mathrm{H}}_{2}}}}\right)}^{-1}+{\left(\frac{{d}_{p,A}}{3}\sqrt{\frac{8RT}{\pi {M}_{{\mathrm{H}}_{2}\mathrm{O}}}}\right)}^{-1}\right)\right)}$$

**Fig. 30:**
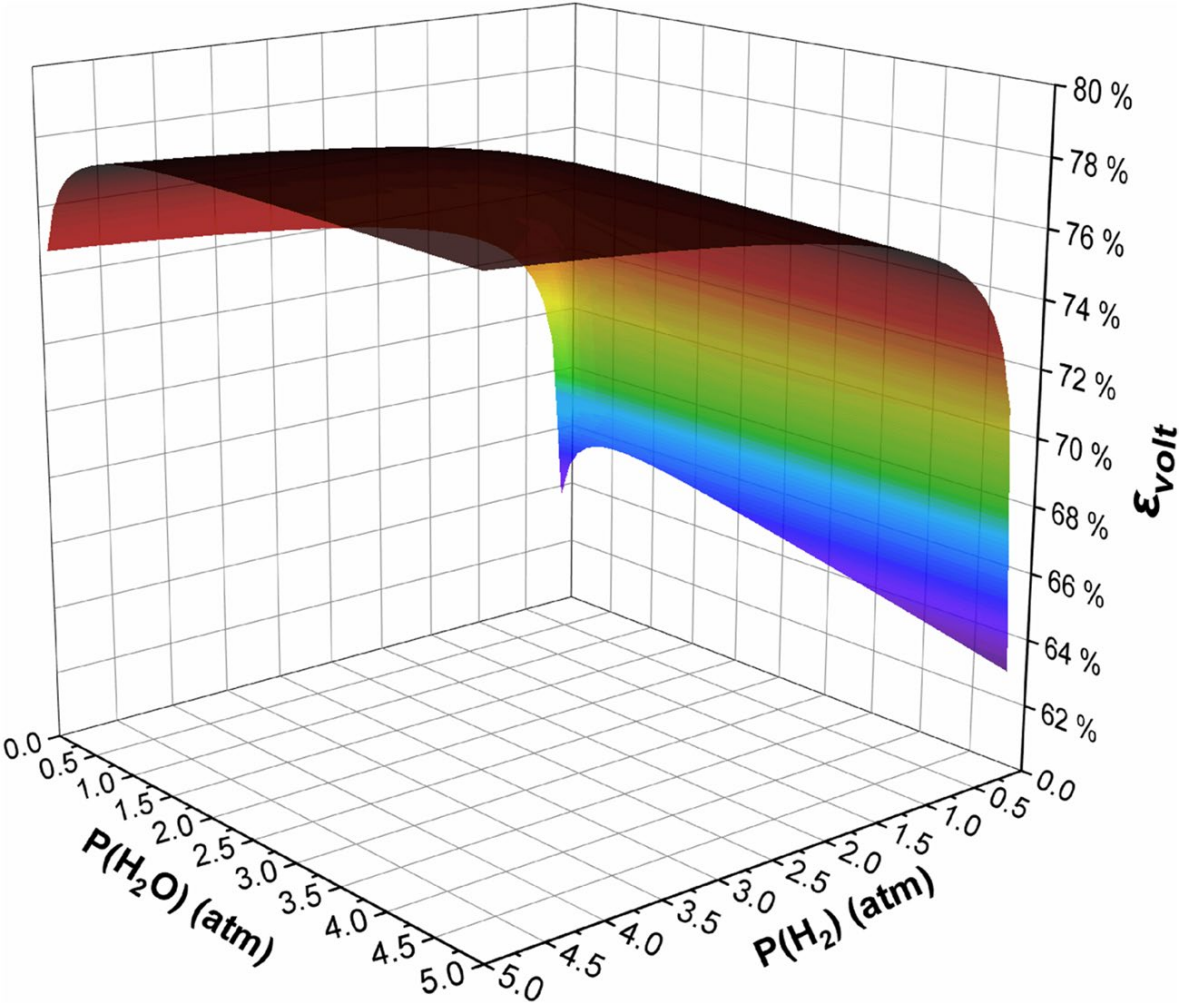
Voltaic efficiency (*ε*_volt_) of a solid oxide fuel cell as a function of *P*(H_2_) and *P*(H_2_O). Parameters of the function are given in Table 4

Combining Eqs. 5, 9–11, and 17 gives the equation for the voltaic efficiency (*ε*_volt_) of a SOFC. Figure 30 shows the influence of hydrogen (*P*_H2_) and water (*P*_H2O_) partial pressure on *ε*_volt_. Since water is a product of the reaction, Le Chatelier’s principle and the Nernst equation predict that a lower *P*_H2O_ will increase the voltage/rate of reaction for the cell. However, Fig. 30 shows that a *P*_H2O_ < 0.5 atm (at the conditions listed in Table 4) will greatly lower the *ε*_volt_ with an exponential decrease in *ε*_volt_ at *P*_H2O_ < 1.5 atm; a *P*_H2O_ > 1.5 atm will linearly decrease *ε*_volt_ but at a very minimal rate. There is an exponential drop in *ε*_volt_ at *P*_H2_ < 1 atm with a more linear increase in *ε*_volt_ above 1 atm; *ε*_volt_ will continue to increase linearly upon increasing *P*_H2_.

**Table 4:** Parameters used to simulate the performance of SOFCs shown in Fig. 30 [177–179]

Variable	Value
*i* (current density)	0.5 A/cm^2^
*R*_ohm_	0.35 Ω cm^2^
*i*_*o,A*_ (anode exchange current density) [177]	0.5 A/cm^2^
*i*_*o,C*_ (cathode exchange current density) [177]	0.3 A/cm^2^
*L*_*A*_ (anode diffusion length) [178]	60 μm
*L*_*C*_ (cathode diffusion length) [178]	30 μm
*d*_*p,A*_ (anode pore diameter)[178]	0.65 μm
*d*_*p,C*_ (cathode pore diameter) [179]	1 μm
*T* (temperature)	973.15 K
*P*_*C*_ (cathode pressure)	1 atm
*P*_O2_ (oxygen partial pressure)	0.21 atm

## Solid oxide fuel cell applications

### Residential cogeneration

**Fig. 31:**
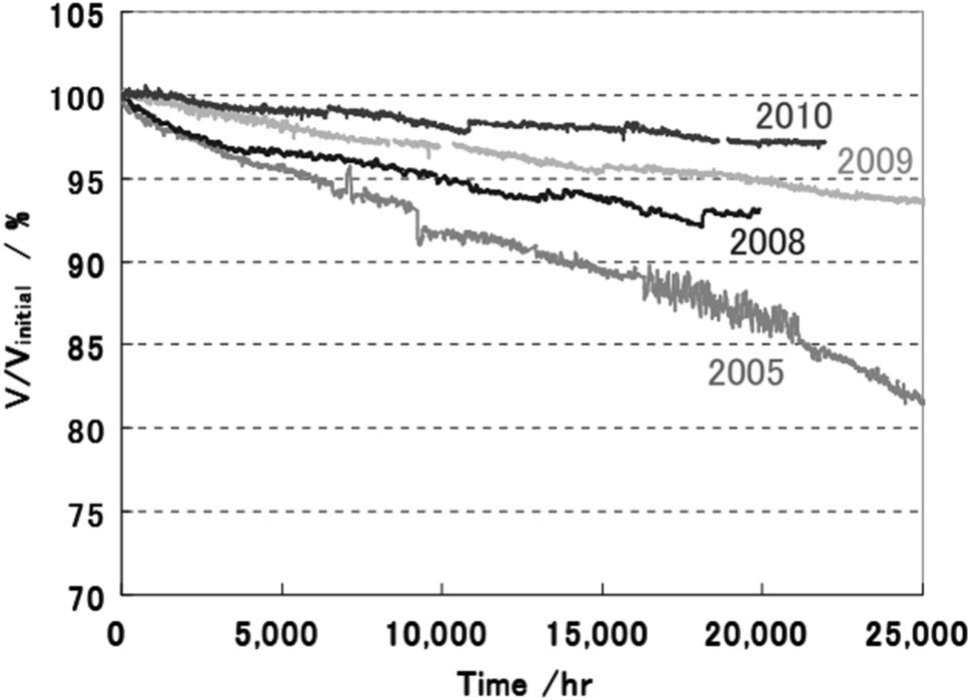
Durability tests of ENE-FARM type-S developed by Osaka Gas [184]

In an ideal SOFC, all the chemical energy present in the feed gases will be converted to electrical energy. However, during operation, some chemical energy is converted to thermal energy mainly due to Joule heating and heat generated by the electrochemical reactions where the HOR is slightly endothermic and the ORR is largely exothermic making the net reaction exothermic [180–182]. The high operating temperature and waste heat produced by the SOFC creates high-temperature exhaust gas which can be utilized for residential/commercial heating. Given natural gas is a primary fuel for residential and commercial heating, many cities have established pipelines to directly supply buildings with natural gas [183]. With the infrastructure already in place, SOFC units can be installed in buildings for dual power and heating. One of the most successful examples of this is the joint development between Osaka Gas, Kyocera, Toyota Motor, and Aisin on the ENE-FARM type S. The Osaka Gas and Kyocera SOFC system showed 45% chemical-to-electrical energy conversion, 30% chemical-to-thermal energy conversion, and 15% of the energy was lost [184]. Over each year, the electrical efficiency of the ENE-FARM type S was improved by thinning the electrolyte, electrodepositing (Zn, Mn, Co)_3_O_4_ on the stainless steel interconnects, improving the airflow, enhancing the thermal insulation, and improving the accuracy of fuel delivery [185, 186]. One of the ENE-FARM type s’s deployed in 2009 has achieved over 10 years of operation with only a 5% drop in chemical-to-electrical energy conversion efficiency (Fig. 31) [187]. The 2016 model features a 0.3 m^3^, 106 kg SOFC/hot water storage tank connected to a 0.1 m^3^, 38 kg hot water unit. It has an estimated lifespan of 10 years and can output a maximum of 700 W (enough to sustain the average home energy demand) with a chemical-to-electrical conversion efficiency of 52%; 35% of the electrical losses are in the form of thermal energy used to heat the hot water unit and the remaining 13% are losses [185].

### Automotive

In 2016, Nissan Motor Co. unveiled the world’s first SOFC-powered vehicle prototype capable of reaching over 600 km on a 30-L bio-ethanol tank [188]. However, electrode or electrolyte-supported cells made of ceramics are damaged upon rapid heating due to poor thermal shock resistance and require slow heating to reach the operating temperature [189]. The slow startup of these cell designs will not work for automotive purposes where the SOFC will need to be quickly heated and cycled between room and operating temperature. By adding a metal support layer (MS), typically composed of ferric stainless steel, cells can withstand larger temperature gradients and faster thermal cycling [190–192]. UK based, Ceres Power has partnered with Bosch, Nissan, and Weichai Power to commercialize MS-SOFCs [193]. Utilizing the Ceres SteelCell® technology, Weichai commercialized the world’s first high-power MS-SOFC running on biomass gas and H_2_ with a chemical-to-electrical conversion efficiency of > 60%, chemical-to-thermal conversion efficiency of 32.55%, with the remaining 7.45% being losses [194, 195]. The potential for these SOFCs to be utilized in vehicles was seen by Nissan and 2018, partnering with Ceres Power and The Welding Institute to develop electric vehicles equipped with SOFCs as range extenders [196].

## Conclusions

SOFCs operating with hydrogen as a fuel are a promising source of carbon–neutral energy that uses a chemical-to-electrical energy conversion process to convert oxygen and hydrogen into electricity and water. For IT-SOFC, the cathode must have high catalytic activity towards the ORR (ASR < 0.1 Ω cm^2^), MIEC (> 100 S/cm), compatibility with electrolyte and interconnect materials, and structural stability under SOFC operating conditions. SOFC cathodes typically have a perovskite, layered-perovskite, or double-perovskite type-structure with perovskite-type structures being more widely used/studied due to their higher stability and conductivity. At ITs, the anode must have high catalytic activity towards the HOR (ASR < 0.1 Ω cm^2^), MIEC (> 100 S/cm), compatibility with electrolyte and interconnect materials, and structural stability under SOFC operating conditions. SOFC anodes are typically Ni-cermet, Cu-cermet, or perovskite-type anodes with Ni-cermet anodes being more widely used/studied due to their superior conductivity, HOR activity, and stability. At ITs, the electrolyte must have high oxide ion conductivity (> 0.1 S/cm) with minimal electronic conductivity (~ 0 S/cm), structural stability under SOFC operating conditions, and compatibility with electrode and interconnect materials. SOFC electrolytes are typically fluorite, perovskite, or apatite-type electrolytes with fluorite-type materials being more popular due to their higher structural stability; perovskite-type electrolytes have been shown to have higher ionic conductivity at lower temperatures than fluorite-type materials but are more reactive and have a larger manufacturing/material cost. At SOFC conditions, the interconnect must be a purely electronic conductor (> 1 S/cm), have a low ASR (< 0.1 Ω cm^2^), stable in reducing and oxidizing environments, have a TEC match with the electrodes and electrolyte, and have no permeability towards oxygen or hydrogen. SOFC interconnects are either ceramic or metal-based with metal-based interconnects being more widely used due to their higher electronic conductivity and lower hydrogen/oxygen permeability.

SOFC efficiency is separated into three components: thermodynamic, fuel, and voltaic efficiency. The thermodynamic efficiency is the ratio between the output energy (Δ*G*) and the input energy (Δ*H*). The fuel efficiency is the ratio between the moles of electrons generated and the moles of fuel supplied; fuel efficiency can be increased using fuel recovery techniques. The voltaic efficiency is the ratio between the operating cell potential (Nernst potential minus the activation, ohmic, and concentration overpotential) and the Nernst potential. It is found that the voltaic efficiency exponentially decreases when  *P*_H2O_ and *P*_H2_ < *P*_O2_ and linearly increases with *P*_H2O_ and *P*_H2_.

The high operating temperature of SOFCs produces high-temperature exhaust gas making SOFCs an appealing option for sectors in need of combined heat and power. Many American states such as Utah and California as well as Canadian provinces such as Alberta or Saskatchewan already supply a large percentage of homes with natural gas for heating; with the infrastructure already in place, the transition towards residential sectors being powered by SOFCs is a realistic goal for the near future. This concept has been successfully deployed in Japan with the ENE-FARM type S being sold and installed since 2020. To make this technology cleaner, homes should be equipped with carbon capture technology to avoid the CO_2_ emissions when utilizing natural gas as a fuel or homes should be directly supplied with hydrogen.

## Data Availability

No datasets were generated or analyzed during the current study.
